# IP_7_-SPX Domain Interaction Controls Fungal Virulence by Stabilizing Phosphate Signaling Machinery

**DOI:** 10.1128/mBio.01920-20

**Published:** 2020-10-20

**Authors:** Desmarini Desmarini, Sophie Lev, David Furkert, Ben Crossett, Adolfo Saiardi, Keren Kaufman-Francis, Cecilia Li, Tania C. Sorrell, Lorna Wilkinson-White, Jacqueline Matthews, Dorothea Fiedler, Julianne Teresa Djordjevic

**Affiliations:** a Centre for Infectious Diseases and Microbiology, The Westmead Institute for Medical Research, Sydney, NSW, Australia; b Sydney Medical School-Westmead, University of Sydney, Sydney, NSW, Australia; c Marie Bashir Institute for Infectious Diseases and Biosecurity, University of Sydney, Sydney, NSW, Australia; d Leibniz-Forschungsinstitut für Molekulare Pharmakologie, Berlin, Germany; e Sydney Mass Spectrometry, University of Sydney, Sydney, NSW, Australia; f Medical Research Council Laboratory for Molecular Cell Biology, University College London, London, United Kingdom; g School of Life and Environmental Sciences, University of Sydney, Sydney, NSW, Australia; University of Georgia

**Keywords:** IP7, inositol pyrophosphate, inositol polyphosphate, SPX domain, cyclin-dependent kinase inhibitor, PHO pathway, Pho81, *Cryptococcus neoformans*, fungal virulence

## Abstract

In the human-pathogenic fungus Cryptococcus neoformans, the inositol polyphosphate signaling pathway is critical for virulence. We recently demonstrated the key role of the inositol pyrophosphate IP_7_ (isomer 5-PP-IP_5_) in driving fungal virulence; however, the mechanism of action remains elusive. Using genetic and biochemical approaches, and mouse infection models, we show that IP_7_ synthesized by Kcs1 regulates fungal virulence by binding to a conserved lysine surface cluster in the SPX domain of Pho81. Pho81 is the cyclin-dependent kinase (CDK) inhibitor of the phosphate signaling (PHO) pathway. We also provide novel mechanistic insight into the role of IP_7_ in PHO pathway regulation by demonstrating that IP_7_ functions as an intermolecular “glue” to stabilize Pho81 association with Pho85/Pho80 and, hence, promote PHO pathway activation and phosphate acquisition. Blocking IP_7_-Pho81 interaction using site-directed mutagenesis led to a dramatic loss of fungal virulence in a mouse infection model, and the effect was similar to that observed following *PHO81* gene deletion, highlighting the key importance of Pho81 in fungal virulence. Furthermore, our findings provide additional evidence of evolutionary divergence in PHO pathway regulation in fungi by demonstrating that IP_7_ isomers have evolved different roles in PHO pathway control in C. neoformans and nonpathogenic yeast.

## Abstract (precis)

Invasive fungal diseases pose a serious threat to human health globally with >1.5 million deaths occurring annually, 180,000 of which are attributable to the AIDS-related pathogen, Cryptococcus neoformans. Here, we demonstrate that interaction of the inositol pyrophosphate, IP_7_, with the CDK inhibitor protein, Pho81, is instrumental in promoting fungal virulence. IP_7_-Pho81 interaction stabilizes Pho81 association with other CDK complex components to promote PHO pathway activation and phosphate acquisition. Our data demonstrating that blocking IP_7_-Pho81 interaction or preventing Pho81 production leads to a dramatic loss in fungal virulence, coupled with Pho81 having no homologue in humans, highlights Pho81 function as a potential target for the development of urgently needed antifungal drugs.

## INTRODUCTION

Cryptococcus neoformans causes fatal meningitis worldwide, especially in immunosuppressed individuals and is responsible for more than 220,000 infections and 180,000 deaths annually (1). Infection is initiated in the lungs and can spread via the blood to the brain to cause meningitis that is fatal without treatment. All fungi, including C. neoformans, use signaling pathways to respond and adapt to host stress and hence to promote their pathogenicity (2). The inositol polyphosphate synthesis pathway, which produces the inositol pyrophosphate 5-PP-IP_5_ (IP_7_) (3–8), and the phosphate sensing and acquisition (PHO) pathway (3, 5) are essential for fungal growth in the lung and spread of infection to the brain. However, whether 5-PP-IP_5_-mediated virulence impairment is due to defects in phosphate homeostasis remains to be addressed.

**FIG 1:**
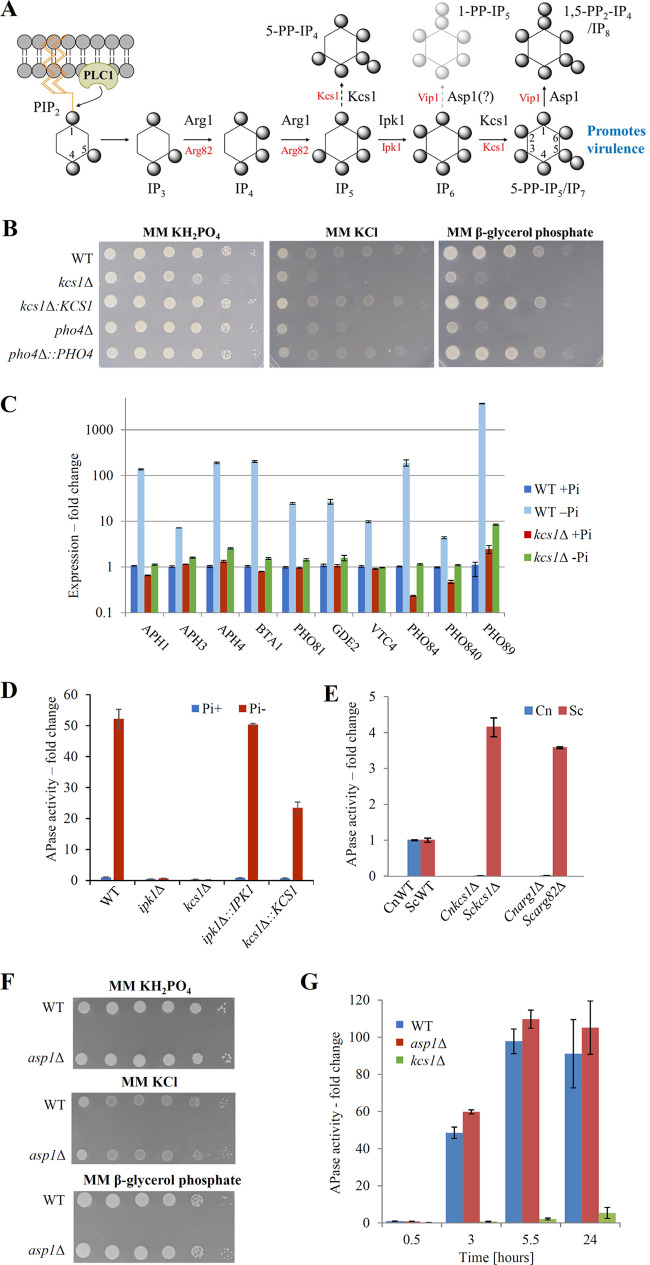
Kcs1-derived 5-PP-IP_5_ is the only inositol polyphosphate required for PHO pathway activation in C. neoformans and has opposing roles in PHO pathway activation in C. neoformans and S. cerevisiae. (A) Inositol polyphosphate biosynthetic pathways in C. neoformans and S. cerevisiae. Spheres represent phosphate groups. Cryptococcal enzymes are indicated in black, and enzymes for S. cerevisiae are indicated in red. In C. neoformans, phospholipase C1 (PLC1)-derived IP_3_ is sequentially phosphorylated to IP_4-5_ and IP_6_ by Arg1 and Ipk1, respectively. Kcs1 generates PP-IP_4_ and 5-PP-IP_5_/IP_7_ from IP_5_ and IP_6_, respectively. However, PP-IP_4_ is only detected in the *ipk1*Δ mutant. Asp1-derived 1,5-PP_2_-IP_4_, but not 1-PP-IP_5_, has been detected in C. neoformans. (B) 5-PP-IP_5_ is required for optimal growth in the absence of phosphate. Overnight YPD cultures were serially diluted (10^6^ to 10^1^ cells per 5 μl) and spotted onto YPD agar. Plates were incubated at 30 and 37°C for 2 days before being photographed. Growth of the 5-PP-IP_5_-deficient C. neoformans mutant strain (*kcs1Δ*) is attenuated to a similar extent as the PHO pathway activation-defective mutant strain (*pho4Δ*). (C) Expression of phosphate-responsive genes regulated by Pho4 is compared by qPCR following growth in the presence and absence of phosphate (calculated using the –ΔΔ*C_T_* method and *ACT1* as the housekeeping gene. The expression in each strain is normalized to the WT +Pi. (D) 5-PP-IP_4_ cannot substitute for 5-PP-IP_5_ in promoting PHO pathway induction since the *ipk1*Δ mutant strain, which accumulates 5-PP-IP_4_, fails to activate the PHO pathway in response to phosphate deprivation. APase activity refers to the extent of *p*-nitrophenyl phosphate hydrolysis by extracellular APases quantified spectrophotometrically at 420 nm (see Materials and Methods for a detailed description). The results are expressed as fold change relative to WT+P_i_. (E) 5-PP-IP_5_ has opposing roles in PHO pathway activation in C. neoformans (Cn) and S. cerevisiae (Sc). PHO pathway activation during phosphate deprivation is compared in WT Cn and Sc and their congenic 5-PP-IP_5_-deficient strains (*arg1Δ/ipk2Δ* and *kcs1Δ*). APase activity was measured as in panel D and normalized to the APase activity of the corresponding WT strains. (F and G) Asp1-derived 1-PP-IP_5_ and 1,5-PP_2_-IP_4_ are dispensable for PHO pathway activation and growth of C. neoformans during phosphate deprivation. A drop dilution test was performed as described previously (see panel B). In panel G, PHO pathway activation was assessed using the APase activity assay and normalized to WT at 0.5 h. All bar graphs represent the means ± the standard deviations of three biological replicates.

As an organism with a haploid genome, C. neoformans served as a useful model to pioneer the characterization of the inositol polyphosphate synthesis pathway in a human fungal pathogen (3–8). Using an inositol polyphosphate kinase (IPK) gene deletion approach to block IP production at different sites, it was shown that the inositol pyrophosphate, 5-PP-IP_5_, is produced by the sequential phosphorylation of inositol trisphosphate (IP_3_) by the IPKs Arg1, Ipk1, and Kcs1 and that 5-PP-IP_5_ is the direct product of Kcs1 (Fig. 1A). In comparison to the other IP products in the pathway, loss of 5-PP-IP_5_ had the most negative impact on virulence in a mouse model (4). 5-PP-IP_5_ is the main IP_7_ isomer in eukaryotic cells and consists of a *myo*-inositol backbone with five covalently attached phosphates and one di(pyro)phosphate at position 5. 5-PP-IP_5_ is further phosphorylated at position 1 by Asp1 to produce 1,5-PP_2_-IP_4_ (IP_8_) (9, 10). Loss of IP_8_ had minimal impact on cellular function and virulence (4). The role of 5-PP-IP_5_ in other human fungal pathogens has not been determined, presumably due to the inability to create viable IPK deletion mutants. However, the creation of a heterozygous *ARG1*/*IPK2* deletion mutant in Candida albicans demonstrated important roles for IPK products in cellular function (11).

Although 5-PP-IP_5_ plays a critical role in fungal virulence, it is unclear how it functions at the molecular level. In nonpathogenic fungi, plants and mammalian cells inositol pyrophosphates, which are highly negatively charged, form electrostatic interactions with the positively charged binding pocket of SPX domains found in components of the phosphate homeostasis machinery (12–20). The term SPX is derived from the proteins in which the domain was first discovered (Syg1, Pho81, and Xpr1). SPX domains are small (135 to 380 residues long). They are either located at the N termini of proteins or occur as independent, single-domain proteins. The interaction of inositol polyphosphates with SPX domains has been shown to modulate phosphate sensing, transport and storage (16, 21).

In fungi, phosphate homeostasis is regulated by the PHO pathway. The mechanism of PHO pathway regulation in the model yeast, Saccharomyces cerevisiae, and in C. neoformans is mostly conserved, except for the absence of a transcriptional coregulator in C. neoformans, which coincides with an expanded number of gene targets (22, 23). In both organisms, phosphate deprivation is sensed by a core regulatory CDK complex comprised of the kinase Pho85, the cyclin Pho80, and the CDK inhibitor (CKI) Pho81, which initiates a transcriptional response aimed at restoring cellular phosphate levels (3, 5, 24, 25). When phosphate is abundant, Pho85 is active and phosphorylates the transcription factor Pho4, thus facilitating its export from the nucleus. When phosphate is scarce, Pho81 inhibits Pho85, preventing Pho4 phosphorylation and its export from the nucleus. This leads to the induction of genes involved in the acquisition of phosphate and potentially other nutrients in the case of C. neoformans (22, 26, 27). Blocking transcriptional activation of the PHO genes in C. neoformans and C. albicans by deleting the Pho4-encoding gene attenuated virulence in a mouse infection model (3, 28). In S. cerevisiae, activation of the PHO pathway requires the Vip1-derived IP_7_ isomer, 1-PP-IP_5_ (29).

In this study, we investigate the role of Kcs1-derived 5-PP-IP_5_ in PHO pathway activation in the fungal pathogen C. neoformans and provide evidence of additional evolutionary divergence in PHO pathway regulation in fungi. We also show that the critical roles of 5-PP-IP_5_ and Pho81 in virulence are conveyed primarily via 5-PP-IP_5_ interaction with the SPX domain of Pho81 and provide novel mechanistic insight into how inositol pyrophosphates regulate PHO pathway activation.

## RESULTS

### Kcs1-derived 5-PP-IP_5_ is required for PHO pathway activation in *C. neoformans*.

The inositol polyphosphate biosynthetic pathway in C. neoformans is represented in Fig. 1A. 5-PP-IP_5_, derived from Kcs1, is the major IP_7_ isomer in fungi. Kcs1 activity is also necessary for the subsequent generation of 1,5-PP_2_-IP_4_ (IP_8_) by Asp1. To determine whether these inositol pyrophosphates play a role in phosphate homeostasis in C. neoformans, growth of the *kcs1Δ* and *pho4Δ* strains was compared in the absence of free phosphate. The results in Fig. 1B demonstrate that growth of both strains is similarly attenuated in either phosphate-free medium (MM-KCl) or in medium where all phosphate is covalently bound to glycerol (β-glycerol-phosphate).

Next, we investigated whether delayed growth of the *kcs1Δ* mutant in the absence of phosphate correlates with an inability to upregulate genes involved in phosphate acquisition (PHO genes). PHO genes in C. neoformans encode three acid phosphatases, including secreted Aph1, which is a biochemical reporter for PHO pathway activation (5, 25); three high-affinity phosphate transporters (Pho84, Pho840, and Pho89) (24); Vtc4 (a component of the Vacuolar Transport Chaperone complex involved in synthesizing inorganic polyphosphate as a phosphate store) (12, 24); two proteins involved in lipid remodeling and phosphate conservation (betaine lipid synthase [Bta1] and glycerophosphodiesterase [Gde2]) (30, 31); and the CDKI, Pho81. Expression of these genes is upregulated in the wild type (WT) following phosphate starvation and is controlled by the transcription factor Pho4 (3, 4, 24, 25). Similar to the *pho4Δ* mutant (3), the PHO genes remained suppressed in the *kcs1Δ* mutant relative to the WT (Fig. 1C), indicating that 5-PP-IP_5_ (the product of Kcs1) and/or its derivative 1,5-PP_2_-IP_4_ (produced by Asp1) are essential for PHO pathway activation and that the precursors of 5-PP-IP_5_ (IP_3_, IP_4_, IP_5_, and IP_6_) play little or no role in the PHO pathway activation.

In a previous study, we showed that the cryptococcal *ipk1Δ* mutant accumulates significant quantities of another inositol pyrophosphate, 5-PP-IP_4_. The *ipk1Δ* mutant is deficient in the native Kcs1 substrate IP_6._ Consequently, Kcs1 phosphorylates IP_5_ at the 5 position to form 5-PP-IP_4_. Using the *ipk1Δ* mutant, we investigated whether 5-PP-IP_4_, which has a similar structure to 5-PP-IP_5_, can also promote PHO pathway activation. Production of extracellular acid phosphatase was used as a reporter to quantify PHO pathway activation in phosphate-starved WT and mutant cells. The results in Fig. 1D demonstrate that, despite its structural similarity to 5-PP-IP_5_ and high abundance in the *ipk1Δ* mutant strain, 5-PP-IP_4_ cannot substitute for the native Kcs1 products in activating the PHO pathway, even though it alleviated some of the *kcs1*Δ-specific phenotypic defects (7).

In contrast to what we observed in C. neoformans (Fig. 1C and D), previous reports in S. cerevisiae suggest that PHO gene expression is constitutively active in the *kcs1*Δ mutant (32). To investigate this further, we assessed PHO pathway activation in WT C. neoformans and S. cerevisiae and their congenic 5-PP-IP_5_-deficient mutant strains (Cn*arg1Δ/*Sc*arg82Δ* and *kcs1Δ*) in parallel. The results in Fig. 1E confirm that the absence of Kcs1-derived inositol pyrophosphates does elicit opposite effects on PHO pathway activation in the two yeast species. Hyperactivation of the PHO pathway in the Sc*kcs1Δ* mutant is consistent with that observed by Auesukaree et al. (32).

### Asp1/Vip1-derived inositol pyrophosphates are dispensable for PHO pathway activation in *C. neoformans* and *S. cerevisiae*.

Asp1 (C. neoformans) and its ortholog Vip1 (S. cerevisiae) phosphorylate 5-PP-IP_5_ to produce 1,5-PP_2_-IP_4_ (4). Vip1 also phosphorylates IP_6_ to produce an alternate isomer of IP_7_, 1-PP-IP_5_. Although we have never detected 1-PP-IP_5_ in WT C. neoformans or in the *kcs1*Δ mutant (4), we considered the possibility that Asp1 produces small quantities of 1-PP-IP_5_ in C. neoformans. To investigate the involvement of 1-PP-IP_5_ and 1,5-PP_2_-IP_4_ in PHO pathway activation in C. neoformans, we employed the *ASP1* deletion mutant (*asp1Δ*). First, we assessed growth of *asp1Δ* on minimal medium (MM) without phosphate and in the presence of β-glycerol-phosphate as the only source of phosphate. Under both conditions, the growth of *asp1Δ* and WT strains was similar (Fig. 1F). This contrasted with the compromised growth observed for the *kcs1*Δ mutant. Next, we quantified PHO pathway activation in WT, *kcs1Δ*, and *asp1Δ* strains using the acid phosphatase reporter assay. Cultures were shifted from phosphate-replete to phosphate-deficient medium and production of secreted acid phosphatase was measured for up to 24 h. Similar to the results shown in Fig. 1C to E, acid phosphatase activity was almost abolished in the *kcs1Δ* mutant over the experimental time course (Fig. 1G). In contrast, acid phosphatase activity in WT and *asp1Δ* strains had increased ∼100-fold by 5.5 h of phosphate deprivation and plateaued out to 24 h. Thus, Kcs1-derived 5-PP-IP_5_, but neither 1-PP-IP_5_ nor 1,5-PP_2_-IP_4_, promotes PHO pathway activation in C. neoformans. Vip1-derived IP_7_ was implicated in PHO pathway activation in S. cerevisiae (29). However, we found phosphate deprivation-induced PHO pathway activation to be comparable in the S. cerevisiae WT and *vip1Δ* mutant (see Fig. S1 in the supplemental material). Overall, the results in Fig. 1 show that, in contrast to S. cerevisiae, Kcs1-derived 5-PP-IP_5_ is the main IPK pathway product involved in PHO pathway activation in C. neoformans and suggest that the PHO pathway has become rewired in C. neoformans.

10.1128/mBio.01920-20.2 FIG S1 Loss of Vip1-derived inositol pyrophosphates in S. cerevisiae does not affect PHO pathway activation. The Sc*arg82*Δ/*arg1*Δ and Sc*kcs1*Δ strains are included for comparison. PHO pathway activation in response to phosphate deprivation was assessed using the acid phosphatase reporter assay. Activity data for mutant strains were normalized to WT. Bars represent the means ± standard deviations (*n* = 3 biological replicates). Download FIG S1, PDF file, 0.02 MB. Copyright © 2020 Desmarini et al. 2020 Desmarini et al. This content is distributed under the terms of the Creative Commons Attribution 4.0 International license.

### 5-PP-IP_5_ acts upstream of CDK Pho85 to promote PHO pathway activation.

During phosphate deprivation, the CKI Pho81 blocks Pho85 kinase activity and hence phosphorylation of the transcription factor Pho4. Pho4 is subsequently retained in the nucleus to induce expression of PHO genes. In humans, yeast, and plants, inositol pyrophosphates interact with the SPX domain of proteins, including the SPX domain of Pho81 in S. cerevisiae (12–14, 16–18, 20, 33, 34). Like ScPho81, Pho81 in C. neoformans also has an SPX domain. We therefore hypothesized that 5-PP-IP_5_ interacts with cryptococcal Pho81 to modulate PHO pathway activation.

As a first step to testing this hypothesis, we used the CDK inhibitor Purvalanol A to bypass Pho81 inhibition (3, 35) and assess whether the PHO pathway can be reactivated in the absence of 5-PP-IP_5_. The results show that even when phosphate is present, Purvalanol A derepresses the PHO pathway in the WT and 5-PP-IP_5_-deficient mutants, including the *kcs1Δ* mutant, but not in the *pho4Δ* control strain, in which PHO pathway activation is blocked downstream of Pho85 (see Fig. S2A and B). Furthermore, we observed a progressive derepression of the PHO pathway up to 50 μM Purvalanol A in WT and *kcs1Δ* strains irrespective of phosphate status with the effect plateauing at 50 μM (see Fig. S2C). These data suggest that 5-PP-IP_5_ functions upstream of Pho85 to inhibit Pho85 kinase activity and promote PHO pathway activation.

10.1128/mBio.01920-20.3 FIG S2 5-PP-IP_5_ functions upstream of CDK Pho85 to promote PHO pathway activation. (A) The PHO pathway is repressed in WT and the *asp1*Δ mutant when phosphate is available, and in the *ipk1*Δ and *kcs1*Δ mutants regardless of phosphate availability. (B) PHO pathway repression in the presence of phosphate is relieved (derepressed) in all strains except for the *pho4*Δ control, by inhibiting Pho85 with the CDK inhibitor Purvalanol A (50 μM). For panels A and B, the results represent the means ± standard deviation (*n* = 3 biological replicates). (C) Irrespective of P_i_ status, a progressive derepression of the PHO pathway is observed in WT and *kcs1Δ* up to 50 μM Purvalanol A with the effect plateauing at 50 μM (*n* = 1 biological replicate). PHO pathway activation was measured using the acid phosphatase reporter assay. Download FIG S2, PDF file, 0.04 MB. Copyright © 2020 Desmarini et al. 2020 Desmarini et al. This content is distributed under the terms of the Creative Commons Attribution 4.0 International license.

### Key IP7-binding residues in SPX domains are conserved in Pho81 homologs from numerous virulent fungi.

**FIG 2:**
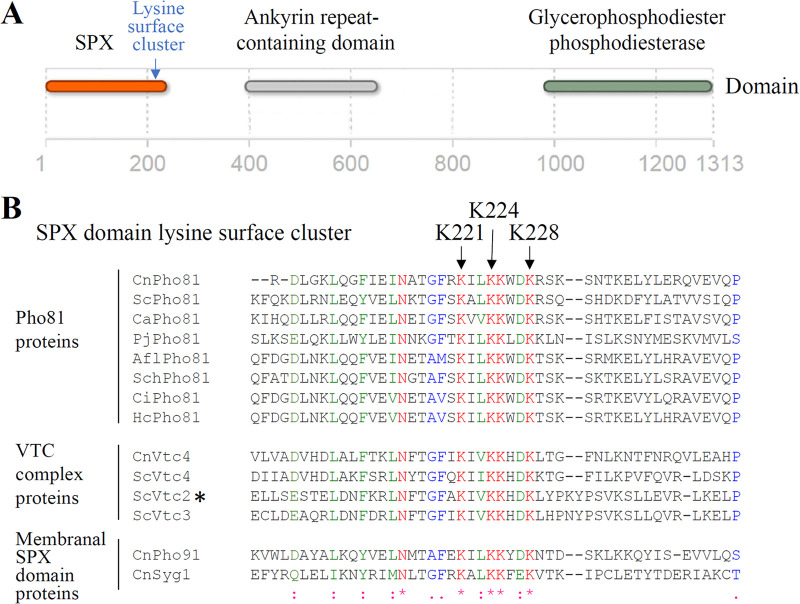
Key IP_7_-binding residues in SPX domains are conserved in Pho81 homologs from numerous virulent fungi. (A) Fungal Pho81 homologues (cryptococcal Pho81 shown as a representative) contain an SPX domain with a lysine surface cluster, an ankyrin repeat domain, and a glycerophosphodiester phosphodiesterase (GDE) domain. (B) Alignment of the SPX domain lysine surface cluster region of CnPho81 and other fungal proteins. A role for the lysine surface cluster in inositol polyphosphate binding has been validated in ScVtc2 (*). Proteins used in the alignment: Cryptococcus neoformans var. *grubii* H99 CnPho81 (XP_012049680), CnSyg1 (XP_012051471), CnPho91 (XP_012049822) phosphate transporter, and CnVtc4 (XP_012049426) vacuolar transporter chaperone 4; Saccharomyces cerevisiae ScPho81 (SGDID:S000003465), ScVtc4 (SGDID:S000003549), ScVtc2 (SGDID:S000001890), and ScVtc3 (SGDID:S000005940); Histoplasma capsulatum HcPho81 (EEH08674); Pneumocystis jirovecii PjPho81 (XP_018228495); Candida albicans CaPho81 (XP_718633); Stachybotrys chartarum SchPho81 (KFA80477); Aspergillus flavus AflPho81 (RAQ58413); and *Coccidioides immitis* CiPho81 (XP_001244784).

Pho81 homologs from numerous fungal species, including C. neoformans and others known to infect humans, contain an N-terminal SPX domain with a lysine surface cluster putatively involved in binding inositol pyrophosphates (Fig. 2A). The SPX domain is followed by an ankyrin repeat domain and a glycerophosphodiester phosphodiesterase domain. The GDE domain in cryptococcal (Cn) Pho81 does not contain critical catalytic residues involved in phospholipid hydrolysis and hence is most likely enzymatically inactive. Alignment of the CnPho81 SPX domain with SPX domains from other fungal proteins, including ScVtc2 for which a role for the basic surface cluster in inositol polyphosphate binding has been validated by site-directed mutagenesis (16), demonstrated the conservation of key lysine residues in CnPho81 (Fig. 2B). We adopted the strategy used by Wild et al. (16) to alter K^221,224,228^ in the cryptococcal Pho81 SPX domain to alanine, creating the Pho81SPX^AAA^ strain to assess the contribution of 5-PP-IP_5_-Pho81 interaction to Pho81 function. The Pho81SPX control strain was taken through the same procedure as Pho81SPX^AAA^ and is therefore genetically identical except for the AAA mutation. As a control, we also deleted the entire *PHO81* gene (*pho81*Δ) (Table 1 ; see also Table S1, Fig. S3, and Fig. S4).

**TABLE 1:** Strains used in this study

Strain	Genotype	Source or reference	Gene identification
C. neoformans			
*pho81Δ*	*pho81Δ*::*HYG*	This study	CNAG_02541
*Δpho81*+*PHO81*	*pho81Δ*::*HYG PHO81-NEO*	This study	CNAG_02541
*spxΔ*	*spxΔ*::*HYG*	This study	Part of CNAG_02541
Pho81^SPX^	*spxΔ*::*HYG NEO-GDE2p-SPX*	This study	CNAG_02541
Pho81^SPXAAA^	*spxΔ*::*HYG NEO-GDE2p-SPX^AAA^*	This study	CNAG_02541
GFP-Pho81	*spxΔ*::*HYG NEO-GDE2p-SPX PHO81-GFP-NAT*	This study	CNAG_02541
GFP-Pho81^SPXAAA^	*spxΔ*::*HYG NEO-GDE2p-SPX^AAA^ PHO81-GFP-NAT*	This study	CNAG_02541
GFP-Pho81	*PHO81-GFP-NAT*	This study	CNAG_02541
GFP-Pho81 *kcs1Δ*	*kcs1Δ*::*NEO PHO81-GFP-NAT*	This study	CNAG_02541/CNAG_02897
*pcl6/7Δ*^*a*^	*pcl6/7Δ*::*NAT*	MKL^*a*^	CNAG_05524
*arg1Δ*	*arg1Δ*::*NEO*	8	CNAG_06500
*kcs1Δ*	*kcs1Δ*::*NEO*	4	CNAG_02897
*kcs1Δ+KCS1*	*kcs1Δ*::*NEO KCS1-NAT*	4	CNAG_02897
*ipk1Δ*	*ipk1Δ*::*NEO*	7	CNAG_01294
*ipk1Δ+IPK1*	*ipk1Δ*::*NEO IPK1-HYG*	7	CNAG_01294
*asp1Δ*	*asp1Δ*::*NEO*	4	CNAG_02161
*pho4Δ*	*pho4Δ*::*NAT*	55	CNAG_06751
*pho4Δ*::*PHO4*	*pho4Δ*::*NAT PHO4+NEO*	3	CNAG_06751
			
S. cerevisiae			
Wild type	*MAT***a** *his31 leu20 met150 ura30* (S288C)	ATCC	BY4741
*arg82Δ*	*arg82Δ*	ATCC	YDR173C
*kcs1Δ*	*MAT***a***/MAT*α *his3Δ1/his3Δ1 leu2Δ0/leu2Δ0 lys2Δ0/met15Δ0/ura3Δ0/ura3Δ0 kcs1Δ*	ATCC	YDR017C
*vip1Δ*	*MAT***a***/MAT*α *his3Δ1/his3Δ1 leu2Δ0/leu2Δ0 lys2Δ0/+ met15Δ0/+ ura3Δ0/ura3Δ0 ylr410w*::*KanMX4*	ATCC	YLR410W

a MKL, Madhani knockout library (http://www.fgsc.net/crypto/crypto.htm) (2015).

10.1128/mBio.01920-20.4 FIG S3 Stepwise construction (A) and verification (B) of the PHO81SPX and PHO81SPX^AAA^ strains and their GFP-tagged counterparts. A detailed description of the process is provided in the supplemental methods above. Primers are listed in Table S1. Download FIG S3, PDF file, 0.3 MB. Copyright © 2020 Desmarini et al. 2020 Desmarini et al. This content is distributed under the terms of the Creative Commons Attribution 4.0 International license.

10.1128/mBio.01920-20.1 TABLE S1 Primers used for all constructs and qPCR. Download Table S1, PDF file, 0.1 MB. Copyright © 2020 Desmarini et al. 2020 Desmarini et al. This content is distributed under the terms of the Creative Commons Attribution 4.0 International license.

10.1128/mBio.01920-20.5 FIG S4 (A) Deletion of the *PHO81* gene using homologous recombination. The Hyg^r^ cassette was used to replace the *PHO81* gene by homologous recombination. (B) Verification of *PHO81* gene deletion and *PHO81* reconstitution by PCR. Genomic DNA was prepared from strain H99 (WT/control), *Δpho81:HYGB* (*Δ*) and *Δpho81+PHO81* (Rec) and specific regions were PCR amplified using internal primer pair (SPX-Seq2/PHO81_3′flank-a), external 5′ primer pair (PHO81_ots s/ActP-a) and external 3′ primer pair (Gal7t-s/PHO81_ots-a). Deletion of the *PHO81* gene (in the *Δ* lane) was confirmed by the absence of the internal region of the *PHO81* locus and the presence of a single band in each PCR amplification across the 5′ (1,067 bp) and 3′ (1,661 bp) recombination junctions. The 5′ (1,067 bp) and 3′ (1,661 bp) amplicons were also present in the Rec strain indicating that insertion of the *PHO81* gene was ectopic. Download FIG S4, PDF file, 0.1 MB. Copyright © 2020 Desmarini et al. 2020 Desmarini et al. This content is distributed under the terms of the Creative Commons Attribution 4.0 International license.

### 5-PP-IP_5_ binding to the Pho81 SPX domain promotes PHO pathway activation.

**FIG 3:**
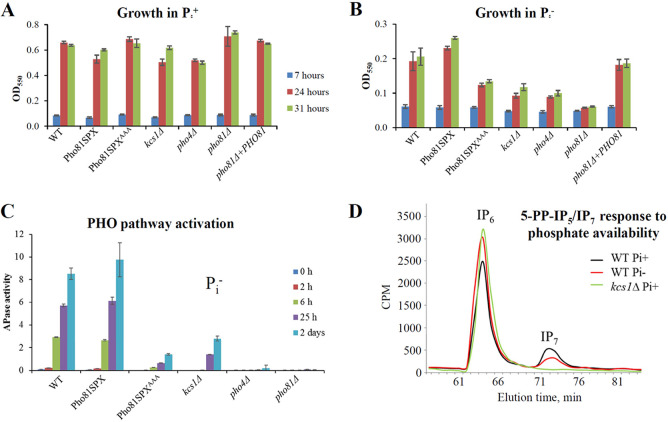
The lysine surface cluster in the Pho81 SPX domain is required for PHO pathway activation. The Pho81SPX^AAA^ and *pho81Δ* strains grow at a rate similar to that of WT, Pho81SPX, and *pho81Δ*+PHO81 strains in the presence (A), but not in the absence (B), of phosphate. In the absence of phosphate, the growth of the Pho81SPX^AAA^ strain is reduced to a level similar to that observed for the *pho81Δ*, *kcs1Δ*, and *pho4Δ* mutant strains. The strains were cultured for 7, 24, and 31 h in MM-KCl, and growth at each time point was assessed by measuring the optical density (550 nm) of the culture using a spectrophotometer. (C) The strains were cultured in MM-KCl, and the PHO pathway activation was assessed at the indicated times using the APase activity assay. APase activity refers to the extent of *p*-nitrophenyl phosphate hydrolysis by extracellular APases quantified spectrophotometrically at 420 nm. In panels A, B, and C, the results represent the means ± the standard deviations of three biological replicates. (D) Comparison of the level of ^3^H-inositol-labeled 5-PP-IP_5_ (IP_7_) in the WT strain by anion-exchange HPLC following growth in P_i_+ or P_i_– medium. The metabolic profile of the *kcs1*Δ strain following growth in YPD medium is provided to indicate the position of IP_7_.

To investigate the role of 5-PP-IP_5_-Pho81 interaction in phosphate homeostasis, growth of the Pho81SPX^AAA^ strain was compared to that of the WT and Pho81SPX control strains in the presence and absence of phosphate (Fig. 3A and B). The *pho81*Δ strain, its reconstituted strain *pho81*Δ*+PHO81*, and the *pho4Δ* and *kcs1Δ* strains were included as controls. All strains had a similar growth rate in the presence of phosphate (Fig. 3A). In contrast, the growth rate of the Pho81SPX^AAA^ and *pho81*Δ mutant strains was reduced relative to that of the WT and Pho81SPX control strains in phosphate-deficient medium (Fig. 3B). As expected, growth of *kcs1Δ* and *pho4Δ* was also reduced in phosphate-deficient medium (Fig. 3B). Next, the role of 5-PP-IP_5_-Pho81 interaction in PHO pathway activation was assessed using an acid phosphatase reporter assay (Fig. 3C). Similar to the growth assays, the PHO pathway activation was abrogated during phosphate deprivation in the Pho81SPX^AAA^, *pho81*Δ, *kcs1Δ*, and *pho4Δ* mutant strains relative to that of the WT and Pho81SPX control strains. 5-PP-IP_5_ levels have been reported to decline in S. cerevisiae in response to phosphate deprivation (16, 36). We now demonstrate that the same occurs in C. neoformans with a decline of approximately ∼50% observed (Fig. 3D. Despite this decline, 5-PP-IP_5_ levels are sufficient to promote PHO pathway activation in WT and Pho81SPX control strains.

**FIG 4:**
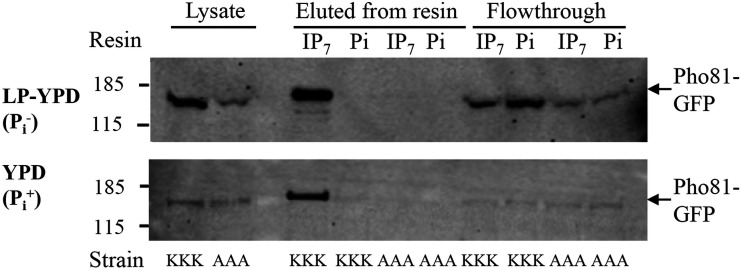
5-PP-IP_5_ interacts with the Pho81 SPX domain via the lysine surface cluster. The GFP-labeled strains were cultured for 5 h in phosphate-depleted medium (LP-YPD) to induce PHO pathway activation or in phosphate-replete (YPD) medium as indicated. Cell pellets were lysed, and the total protein was adjusted to 8 mg/ml to correct for growth differences. Lysates were incubated with 5-PCP-IP_5_-conjugated (IP_7_) and phosphate (P_i_)-conjugated (control) resin. Bound Pho81 from each strain was then compared by SDS-PAGE and anti-GFP Western blotting. Then, 10-μl portions of protein-adjusted lysates prepared from Pho81SPX-GFP (KKK) and Pho81SPX^AAA^-GFP (AAA), respectively, were run as controls. Adjacent lanes (from left to right) contain what was eluted from each resin by the LDS loading buffer and what was present in the flowthrough.

We also confirmed that Pho81 associates with 5-PP-IP_5_ via K^221,224,228^ in the SPX domain by performing affinity capture experiments using a 5-PP-IP_5_-conjugated resin. To enable Pho81 detection by Western blotting, we added a green fluorescent protein (GFP) tag at the C terminus of WT and mutant Pho81 (see Fig. S3) and confirmed that tag addition did not affect functionality (see Fig. S5). The Pho81-GFP expressing strains were cultured in phosphate (P_i_)-deficient and P_i_-replete medium. Cell lysates were incubated with chemically synthesized affinity capture resins, presenting either a stable nonhydrolyzable version of 5-PP-IP_5_ (5-PCP-IP_5_) (37) or P_i_ (as a control), to pull down Pho81SPX-GFP and Pho81SPX^AAA^-GFP. The extent of binding of native and mutant Pho81 proteins (molecular mass, 170.2 kDa) to each resin was compared by anti-GFP Western blotting (Fig. 4). Levels of Pho81SPX and Pho81SPX^AAA^ were more comparable in P_i_-grown versus P_i_-starved cells. Given that the protein concentration was similar in all lysates, increased Pho81SPX relative to Pho81SPX^AAA^ in P_i_-starved cells is attributable to *PHO81* being a phosphate-responsive gene and the PHO pathway being functional only in the Pho81SPX strain (Fig. 1B). Hence, Pho81-mediated inhibition of Pho85 drives its own induction during P_i_ starvation. The affinity capture results demonstrate that under both growth conditions, native Pho81 binds to the 5-PCP-IP_5_, but not to the P_i_, resin. In contrast, the mutated variant does not bind to either resin but appears in the flowthrough. Thus, native Pho81SPX protein, but not its Pho81SPX^AAA^ variant, binds 5-PP-IP_5_.

10.1128/mBio.01920-20.6 FIG S5 Functional verification of the Pho81SPX-GFP-fusion protein. PHO pathway activation (induction of the *APH1* gene) was assessed by qPCR following growth of the Pho81SPX-GFP (WT) and Pho81SPX^AAA^-GFP strains in the presence and absence of P_i_. Gene expression was quantified using the ΔΔCT method using *ACT1* as the house-keeping gene. Similar to the Pho81SPX^AAA^ mutant strain (Fig. 3C), the Pho81SPX^AAA^-GFP mutant strain remains defective in PHO pathway activation. Download FIG S5, PDF file, 0.01 MB. Copyright © 2020 Desmarini et al. 2020 Desmarini et al. This content is distributed under the terms of the Creative Commons Attribution 4.0 International license.

In S. cerevisiae, Pho81 forms a stable complex with Pho85-Pho80 independently of phosphate status, but only inhibits the CDK during phosphate deprivation (38). Interaction of Pho81 with Pho85-Pho80 is primarily via Pho80 (38, 39). To determine whether the association of CDK components in C. neoformans is phosphate dependent, the WT strains expressing either Pho81-GFP (see Fig. S3) or Pho85-mCherry were cultured in P_i_-depleted and P_i_-replete media. GFP trap and an anti-mCherry antibody were used to immunoprecipitate Pho81 and Pho85, respectively, and any associated proteins from cell lysates. CDK components were separated by SDS-PAGE and identified by one-dimensional liquid chromatography-mass spectrometry (1D-LC-MS). In both sets of immunoprecipitations, Pho81, Pho85, Pho80 and a second cyclin, glycogen storage control protein (CNAG_05524), were consistently detected in the CDK complex regardless of phosphate availability (Table 2). A BLAST search against the S. cerevisiae genome database using the glycogen storage control protein sequence as a query revealed that this cyclin is most similar to cyclins Pcl6 and Pcl7 which, among other cyclins, are most closely related to Pho80 (see Fig. S6A). Thus, we renamed this cyclin CnPcl6/7.

**TABLE 2:** CDK components consistently detected in the Pho81SPX-GFP and Pho85-mCherry immunoprecipitations regardless of phosphate status^*a*^

Accession no.	GFP-labeled Pho81						mCherry-labeled Pho85					
	Pi depleted			Pi replete			Pi depleted			Pi replete		
	PEP	% Cov	PSMs	PEP	% Cov	PSMs	PEP	% Cov	PSMs	PEP	% Cov	PSMs
CNAG_01922 (Pho80)	74.52	43.42	46	55.16	33.33	55	87.03	38.16	46	99.93	43.42	50
CNAG_02541 (Pho81)	379.78	40.67	540	331.41	34.50	520	609.66	53.08	347	487.34	37.09	278
CNAG_08022 (Pho85)	138.83	47.32	137	104.12	35.20	143	188.63	51.52	147	176.39	37.76	152
CNAG_05524 (Pcl6)	152.28	62.39	112	83.81	37.97	29	181.42	65.10	113	210.59	55.70	99

a Anti-GFP-Pho81 or anti-mCherry-Pho81 immunoprecipitations were separated by SDS-PAGE and the associated CDK components were identified by 1D-LC-MS. All CDK components (Pho81, Pho85, and Pho80) and an additional cyclin (Pcl6) were detected consistently in both sets of immunoprecipitations prepared from cells grown in phosphate-replete and phosphate-depleted medium. Control immunoprecipitations were also performed on the WT (no GFP or mCherry) and the absence of all CDK components was confirmed. The PEP score (PEP) is based on the probability of identification: scores above 3 are equivalent to a q-value of <0.002. “% Cov” is the percent coverage of the open reading frame the observed peptides match, while the number of peptide spectral matches (PSMs) is proportional to the protein abundance. All PSMs were filtered to ensure a <1% false discovery rate.

10.1128/mBio.01920-20.7 FIG S6 (A) Clustering analysis summarizing cyclin similarity in S. cerevisiae and C. neoformans. Seven cyclins were identified in C. neoformans using a BLAST search. Alignment of the cyclin sequences from C. neoformans and S. cerevisiae using ClustalW (https://npsa-prabi.ibcp.fr/cgi-bin/npsa_automat.pl?page=/NPSA/npsa_clustalw.html), and clustering analysis showed that cryptococcal glycogen storage control protein (renamed CnPcl6/7) clusters with ScPcl6/ScPcl7, is most similar to Pho80 and is 16%/18% identical and 25%/18% similar to ScPcl6/ScPcl7, respectively. (B) Genes encoding CDK components found to be associated by 1D-LC-MS are P_i_ responsive. Overnight YPD cultures of WT H99 were pelleted by centrifugation and cultured for 3 h in minimal medium with P_i_ (MM-KH_2_PO_4_) or without P_i_ (MM-KCl). An OD_600_ of 20 of each culture was pelleted by centrifugation and snap-frozen in liquid nitrogen. RNA was extracted for cDNA synthesis. The expression of *PHO81*, *PHO85*, *PHO80*, and *PCL6/7* was quantified by qPCR using the housekeeping gene, *ACT1*, as a control. The expression is shown as the fold change (induction) compared to the repressing condition with P_i_ (MM-KH_2_PO_4_). The results of a one-way ANOVA Tukey-Kramer multiple-comparison test are shown, with *PHO81* being the most prolific responder to low P_i_. (C) Cyclin Pcl6/7 is not essential for PHO pathway activation in C. neoformans. YPD overnight cultures of the strains indicated were prepared and PHO pathway activation induced by resuspending the cell pellets in inducing (P_i_-deficient) medium (MM-KCl) at an OD_600_ of 1. Noninduced controls were resuspended in P_i_-containing medium (MM-KH_2_PO_4_). The cultures were incubated at 30°C for 3 h prior to performing the APase assay at 37°C for 10 min. APase-mediated hydrolysis of pNPP was quantified spectrophotometrically at 420 nm. Any growth differences among the strains were corrected by measuring the OD_600_ prior to performing the assay. The APase activity was calculated as OD_420_/OD_600_. The *pho81*Δ and *pho81Δ*+*PHO81* strains are included as controls. The results represent the mean of three biological replicates ± the standard deviations. Download FIG S6, PDF file, 0.1 MB. Copyright © 2020 Desmarini et al. 2020 Desmarini et al. This content is distributed under the terms of the Creative Commons Attribution 4.0 International license.

Of all the genes encoding CDK complex components, *PHO81* was the most phosphate responsive (∼14-fold induction) (Fig. 1C; see also Fig. S6B), followed by *PHO80* and *PLC6/7* (∼4- to ∼5-fold induction) (see Fig. S6B). A small increase in *PHO85* gene expression (∼1.8-fold) was observed but was not statistically significant. Although *PCL6/7* is phosphate-responsive, it is dispensable for PHO pathway activation as assessed using a *pcl6/7*Δ mutant (see Fig. S6B). Given its similarity to cyclin homologues involved in glycogen storage in S. cerevisiae, we investigated whether cryptococcal Plc6/7 also has a role in glycogen storage. The results in Fig. S8 demonstrate reduced glucose induction of the glycogen metabolic genes, *GSY2*/CNAG_04621 and *GLC3*/CNAG_00393, in the *pcl6/7*Δ, Pho81SPX^AAA^, and *pho81*Δ strains relative to the WT. The results suggest that, in addition to activating the PHO pathway by interacting with Pho80-Pho85, 5-PP-IP_5_-Pho81 modulates glycogen storage by interacting with Pcl6/7-Pho85.

10.1128/mBio.01920-20.8 FIG S7 Induction of genes involved in glycogen storage is impaired in the *pcl6/7*Δ, PhoSPX^AAA^, and *pho81*Δ strains in response to low glucose. The strains were grown in high (2%)- and low (0.2%)-glucose YPD medium overnight at 30°C to suppress and induce, respectively, the induction of two genes involved in glycogen metabolism: glycogen synthase (*GSY2/*CNAG_04621) and glycogen branching enzyme (*GLC3/*CNAG_00393). An OD_600_ of 20 of each culture was pelleted by centrifugation and snap-frozen in liquid nitrogen. RNA was extracted for cDNA synthesis and quantification of gene expression by qPCR. The primers used for qPCR are listed in Table S1. The induction of both genes in low glucose was normalized to that of WT basal expression in high glucose (not shown) and expressed as a fold change. Results represent the means ± the standard deviations of three biological replicates. The statistical significance was calculated using an unpaired *t* test with Welch’s correction. Download FIG S7, PDF file, 0.02 MB. Copyright © 2020 Desmarini et al. 2020 Desmarini et al. This content is distributed under the terms of the Creative Commons Attribution 4.0 International license.

10.1128/mBio.01920-20.9 FIG S8 Basal *PHO81* expression (in the presence of P_i_) is unaffected by either mutation in the 5-PP-IP_5_-binding site (A) or 5-PP-IP_5_-deficiency in the *kcs1*Δ mutant background (B). (A) Overnight YPD cultures of each strain were pelleted by centrifugation. Pellets were washed twice with water and the cells were used to seed 10 ml of either YPD (Pi+) or MM-KCl (Pi–) to an OD_600_ of 1. Growth was allowed for 3 h. In panel B, strains were grown in YPD overnight. In both panels A and B, cells were pelleted by centrifugation and snap-frozen in liquid nitrogen. RNA was extracted for cDNA synthesis. *PHO81* gene expression was quantified by qPCR using the ΔΔ*C_T_* method, and *ACT1* was used as the house-keeping gene. In panel A, *PHO81* expression is shown as a fold change compared to WT grown in YPD (Pi+). *, No significant difference (NS) relative to Pho81SPX Pi+ (*P* = 0.0899) and WT Pi+ (*P* = 0.9918), as determined by a one-way ANOVA and the Tukey-Kramer multiple-comparison test. In panel B, *PHO81* expression in *kcs1*Δ is shown as a fold change relative to WT, and the difference is not significant (*P* = 0.1261), as determined by an unpaired *t* test with Welch’s correction. In both experiments, the results represent the mean of three biological replicates ± the standard deviations. Download FIG S8, PDF file, 0.1 MB. Copyright © 2020 Desmarini et al. 2020 Desmarini et al. This content is distributed under the terms of the Creative Commons Attribution 4.0 International license.

### PP-IP_5_-Pho81 interaction stabilizes the CDK complex of the PHO pathway.

**FIG 5:**
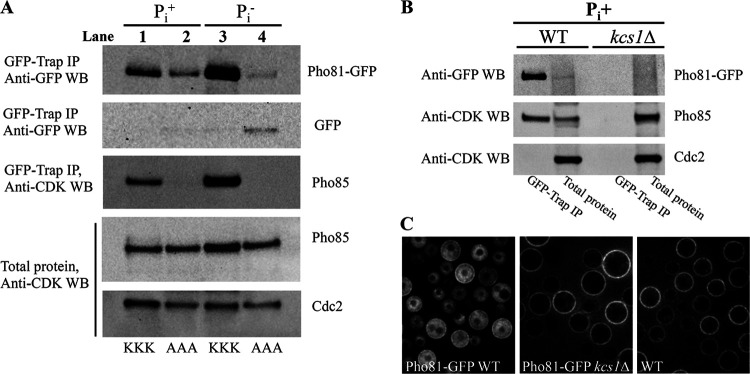
5-PP-IP_5_-Pho81 interaction stabilizes the CDK complex of the PHO pathway. (A) GFP-trap was used to immunoprecipitate Pho81SPX-GFP (KKK, lanes 1 and 3) and Pho81SPX^AAA^-GFP (AAA, lanes 2 and 4) from lysates following cell growth in P_i_+ and P_i_– medium. Immunoprecipitates and total cell lysates (control) were resolved by SDS-PAGE. Immunoprecipitated Pho81-GFP was detected by anti-GFP Western blotting. Anti-CDK antibody, which detects the PSTAIR motif, was used to detect Pho85 in the immunoprecipitates and cell lysates, as well as Cdc2 in the cell lysate, as indicated. The blot is representative of three biological replicates where, on average, Pho85/Pho81SPX^AAA^ association was 2.7-fold weaker than Pho85/Pho81SPX association in P_i_+ cultures. (B) GFP-trap was used to immunoprecipitate Pho81SPX-GFP from WT and *kcs1*Δ lysates following cell growth in P_i_+ medium. Immunoprecipitates and total cell lysates (control) were resolved by SDS-PAGE. Pho81-GFP, Pho85, and Cdc2 were detected by Western blotting as in panel A. (C) Pho81SPX-GFP is not detected by fluorescence microscopy (DeltaVision) in an IP_7_-deficient (*kcs1*Δ) background following cell growth in P_i_+ medium (using the same conditions as in panel B). Autofluorescence of the cell walls is detected in all samples due to the prolonged exposure essential for observing Pho81-GFP.

To investigate whether 5-PP-IP_5_ interaction with Pho81 affects Pho81 association with the CDK complex, Pho81SPX-GFP and Pho81SPX^AAA^-GFP were immunoprecipitated from cells cultured in the presence and absence of P_i_. Pho81-associated Pho85 was then quantified by Western blotting (Fig. 5A). Cdc2 in cell lysates was used as an indicator of sample protein concentration prior to immunoprecipitation. Under P_i_-depleted conditions, Pho81SPX and Pho85 abundance increased to a similar extent (∼2-fold) compared to their levels in cultures supplied with P_i_ (Fig. 5A, compare lanes 1 and 3), consistent with increased CDK complex formation. Increased Pho85 and Pho81 abundance following P_i_ deprivation correlated with increased *PHO85* and *PHO81* gene expression (see Fig. S6B: ∼1.8-fold for *PHO85* and ∼14-fold for *PHO81*). However, the increase in *PHO81* expression far exceeded the increase in Pho81 protein in the immunoprecipitates, consistent with translation of only a proportion of *PHO81* transcripts and/or rapid degradation of excess free Pho81.

Quantification of Pho85 association with native and mutant Pho81 in the absence of PHO pathway activation (P_i_ + culture) demonstrated weaker Pho85 binding to mutant Pho81 (Fig. 5A, compare lanes 1 and 2), suggesting that 5-PP-IP_5_ is required for stabilizing the CDK complex. Interestingly, we observed that the abundance of Pho81SPX^AAA^ declined during P_i_ deprivation/PHO pathway activation (Fig. 5A, compare lanes 2 and 4), rendering comparison of Pho85 association with WT and mutant Pho81 during P_i_ deprivation unfeasible. Using qPCR, we ruled out reduction of *PHO81SPX*^AAA^ gene expression under inducing conditions as a possible explanation (see Fig. S8). Rather, the detection of cleaved GFP in the mutant sample (Fig. 5A, lane 4) was indicative of Pho81 degradation during P_i_ deprivation. The reduced stability of mutant Pho81 under these conditions coincides with lower levels of IP_7_ (Fig. 3D).

To further investigate the impact of 5-PP-IP_5_ interaction with Pho81 on CDK association, we tagged Pho81SPX with GFP in the *kcs1*Δ mutant background and repeated the immunoprecipitations on P_i_+ cultures. Pho81SPX protein was not detected in *kcs1*Δ lysates (total protein) and immunoprecipitations (GFP-Trap IP) (Fig. 5B) or in intact 5-PP-IP_5_-deficient cells by fluorescence microscopy (Fig. 5C). Once again, qPCR ruled out reduced *PHO81* gene expression as a possible explanation (Fig. 1C; see also Fig. S8, using GFP strains and growth conditions identical to those in Fig. 5B). Hence the results are consistent with degradation of Pho81, but not Pho85, in a 5-PP-IP_5_-deficient environment.

**FIG 6:**
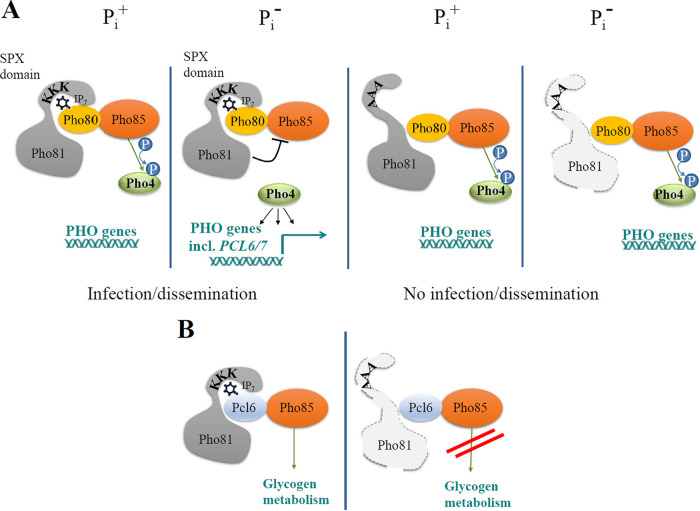
Model depicting the role of 5-PP-IP_5_ in CDK stability and PHO pathway activation in C. neoformans. The binding of 5-PP-IP_5_ to the SPX domain of Pho81 in C. neoformans promotes Pho81 association with Pho80-Pho85 (A) and Pcl6/7-Pho85 (B). 5-PP-IP_5_, which is negatively charged, forms electrostatic interactions with the lysine surface cluster in the SPX domain of native Pho81 and with unidentified basic residues in cyclins Pho80 and Pcl6/7 and therefore stabilizes each CDK complex irrespective of phosphate status. In panel A, 5-PP-IP_5_-bound Pho81 inhibits Pho85 during phosphate deprivation, preventing phosphorylation of Pho4 and triggering PHO pathway activation to promote pathogenicity. In contrast, 5-PP-IP_5_ binding-defective Pho81 cannot form a stable complex with Pho80-Pho85 and Pho85 remains active, phosphorylating Pho4 to prevent PHO pathway activation. In panel B, 5-PP-IP_5_-bound Pho81 may also regulate Pcl6-Pho85 to fine-tune glycogen metabolism. In both panels A and B, 5-PP-IP_5_ binding-defective Pho81 is unstable and becomes degraded.

From the results in Fig. 4 and 5, we propose a model (Fig. 6) where Pho81 stability and association with Pho85-Pho80 and Pho85-Pcl6/7 depends on its ability to bind 5-PP-IP_5_ and where 5-PP-IP_5_-Pho81 interaction promotes PHO pathway activation and glycogen biosynthesis.

### 5-PP-IP_5_-Pho81 interaction is critical for fungal virulence and dissemination.

To determine the impact of 5-PP-IP_5_–Pho81 interaction on cryptococcal virulence, we investigated whether the PHO pathway activation-defective Pho81SPX^AAA^ and *pho81*Δ mutant strains retained key virulence traits characteristic of C. neoformans (e.g., ability to grow at 37°C and produce capsule and melanin). We found that all phenotypes were identical to that of the Pho81SPX, WT, and *pho81*Δ*+PHO81* strains (results not shown).

**FIG 7:**
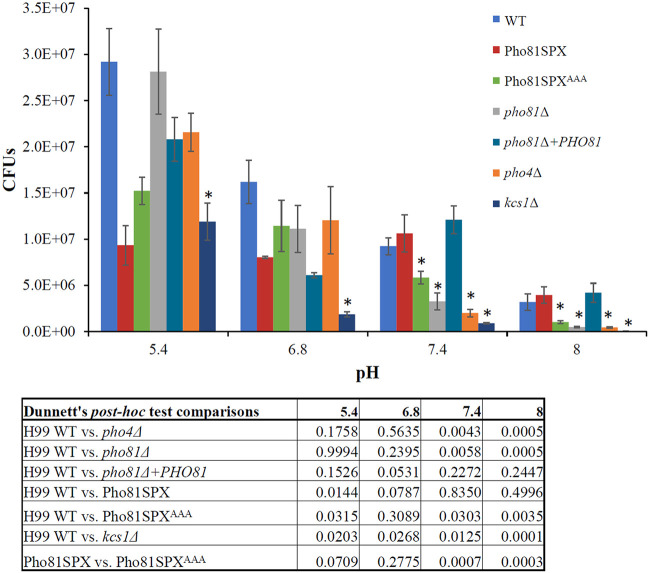
5-PP-IP_5_-Pho81 interaction is required for fungal growth at alkaline pH. The strains indicated were cultured in minimal media containing 1 mM KH_2_PO_4_ with the pH adjusted to 6.8, 7.4, or 8.0 with HEPES buffer and to pH 5.4 with MES buffer. Growth was assessed after 24 h with shaking by quantitative culture (CFU). Results represent the means ± the standard deviations of three biological replicates. Statistical analysis was performed using one-way ANOVA. With the exception of kcs1Δ*, none of the Dunnett’s *post hoc* test comparisons at pH 5.4 and 6.8 were statistically significant relative to their control strain (*P* ≥ 0.05). However, at pH 7.4 and pH 8, growth of the *pho4Δ*, *pho81Δ*, *kcs1Δ*, and Pho81SPX^AAA^ strains was reduced relative to the WT, Pho81SPX, and *pho81Δ+PHO81* strains (*, *P* < 0.05), consistent with the alkaline pH environment mimicking phosphate deprivation.

Despite the availability of significant levels of free phosphate in most environments within the mammalian host, the alkaline pH of host blood and tissues mimics phosphate starvation, leading to activation of the fungal PHO pathway (3, 40, 41). Consistent with this, the PHO pathway activation-defective cryptococcal strain, *pho4Δ*, exhibits reduced growth at alkaline (including host) pH, even when phosphate is available (3). We therefore compared growth of the PHO pathway activation defective Pho81SPX^AAA^ strain and the Pho81SPX control strain at acidic and basic pH and included the WT, *pho4*Δ, *kcs1*Δ, *pho81*Δ, and *pho81*Δ*+PHO81* strains as additional controls (Fig. 7). At pH 5.4 and pH 6.8, none of the pairwise growth differences relative to the parent strain were statistically significant except for the WT versus the *kcs1*Δ strain. The reduced growth of *kcs1*Δ is expected since this mutant grows slower than the WT under nonstress conditions (YPD medium) due to Kcs1 having a pleiotropic role in cellular function (4). In contrast, at pH 7.4 and pH 8 (P_i_+), growth of the *pho4Δ*, *pho81Δ*, *kcs1Δ*, and Pho81SPX^AAA^ strains was reduced relative to the WT, Pho81SPX, and *pho81Δ+PHO81* strains (Fig. 7) consistent with the alkaline pH environment mimicking phosphate deprivation (3, 40).

**FIG 8:**
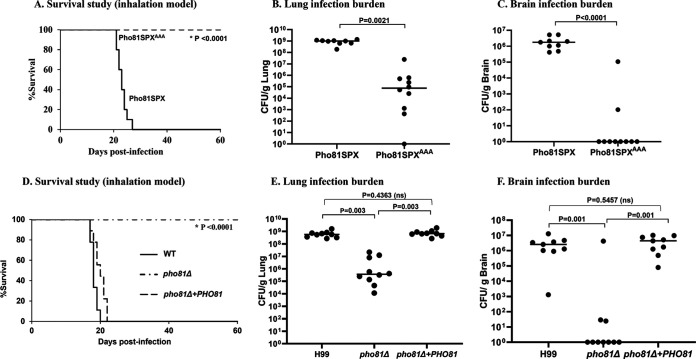
5-PP-IP_5_-Pho81 interaction is critical for fungal virulence and dissemination in a mouse infection model. Mice were infected intranasally with 2 × 10^5^ Pho81SPX or Pho81SPX^AAA^ cells (A) or WT, *pho81*Δ, or *pho81*Δ*+PHO81* cells (D), and their health was monitored for up to 60 days. Infection burdens in the lung (B and E) and brain (C and F) were determined at time of death (Pho81SPX-, WT-, and *pho81*Δ*+PHO81*-infected mice) and at 60 days postinfection (Pho81SPX^AAA^ and *pho81*Δ-infected mice). Lungs and brains were homogenized, serially diluted, and plated onto agar plates. Plates were incubated at 30°C for 2 days. Colony counts were adjusted to reflect CFU per gram of tissue. The difference in survival (log-rank test) and organ burden (Mann-Whitney U test/two-paired *t* test) between Pho81SPX- or Pho81SPX^AAA^-infected groups is statistically significant (i.e., *P* ≤ 0.0021 in all cases). No difference in survival and organ burden was observed between the WT and *pho81*Δ+*PHO81* infection groups. However, the reductions in survival and organ burden observed for the *pho81*Δ-infected mice, relative to the two control strains, was statistically significant (i.e., *P* ≤ 0.003 in all cases).

Next, we assessed what effect blocking 5-PP-IP_5_-Pho81 interaction had on fungal virulence in a mouse inhalation model, which mimics the natural route of infection in humans. All mice infected with the Pho81SPX control strain succumbed to infection with the median survival time being 23 days (Fig. 8A). In contrast, no mice infected with the Pho81SPX^AAA^ mutant became ill, and by 60 days postinfection their average weight had increased by 20 ± 5.5% relative to their average preinfection weight. Organ burdens determined in Pho81SPX-infected mice at time-of-death and in Pho81SPX^AAA^-infected mice at 60 days postinfection show almost no infection in the lungs and brain of Pho81SPX^AAA^-infected mice by 60 days postinfection (Fig. 8B and C). This is consistent with the inability of this strain to establish a lung infection and disseminate to the brain.

We also investigated the effect of deleting the *PHO81* gene on fungal virulence and included the *pho81*Δ*+PHO81* strain as a control (Fig. 8). For the survival analysis, the *pho81Δ* mutant strain behaved similarly to the Pho81SPX^AAA^ strain, with no *pho81Δ-*infected mice succumbing to infection over the 60-day time course (Fig. 8D). Furthermore, the *pho81Δ-*infected mice had gained a similar amount of weight by 60 days postinfection as the Pho81SPX^AAA^-infected mice. As expected, *pho81*Δ*+PHO81*-infected mice had a similar median survival time to that of WT-infected mice. Organ burdens were also determined in WT- and *pho81*Δ*+PHO81*-infected mice at time-of-death and in *pho81Δ-*infected mice at 60 days postinfection. Similar to what was observed for the 5-PP-IP_5_-binding defective strain, the lung and brain burdens were reduced substantially in *pho81Δ-*infected mice relative to both WT- and *pho81*Δ*+PHO81*-infected mice (Fig. 8E and F), consistent with the inability of this strain to establish a lung infection and disseminate to the brain.

## DISCUSSION

Our work has shown that the inositol polyphosphate biosynthesis pathway in C. neoformans intersects with the PHO pathway signaling machinery via Kcs1-derived 5-PP-IP_5_ rather than via Asp1/Vip1-derived 1-PP-IP_5_, providing evidence of evolutionary rewiring with respect to inositol pyrophosphate regulation of the PHO pathway. We also show that 5-PP-IP_5_ exerts much of its effect on virulence by promoting PHO pathway activation via its interaction with the SPX domain of Pho81.

Using crystallographic, biochemical, and genetic analysis, Wild et al. demonstrated that recombinant SPX domains from yeast, filamentous fungal, plant, and human proteins bind 5-PP-IP_5_, IP_6_, and IP_8_ with high affinity but not IP_3_/IP_4_/IP_5_ or free orthophosphate. These researchers also identified conserved lysine residues responsible for PP-IP binding. Substituting these lysine residues with alanine did not impact secondary or tertiary structure of SPX domains but did abrogate PP-IP binding (16). By adopting the same approach and incorporating the same alteration into the SPX domain of the full-length protein, we now extend these findings to Pho81 in C. neoformans, demonstrating that mutation of the conserved lysine residues prevents Pho81 from binding to 5-PP-IP_5_.

From our investigation of CDK component association by 1D-LC-MS and Western blotting, we propose a model where Pho81 association with Pho85-Pho80 depends on 5-PP-IP_5_ interaction with the Pho81 SPX domain and where 5-PP-IP_5_-Pho81 interaction promotes PHO pathway activation (Fig. 6). 5-PP-IP_5_ therefore has a bridging role by promoting the association of CDK complex components, irrespective of phosphate status. Although phosphate deprivation coincided with a decline in 5-PP-IP_5_ levels, more CDK complex formation was observed (Fig. 5, lane 3), suggesting that the levels of 5-PP-IP_5_ under these conditions were sufficient to promote increased CDK complex formation. Interestingly, we found that mutant Pho81 became unstable during P_i_ deprivation (Fig. 5A, lane 4). This could be attributable to 5-PP-IP_5_ stabilizing Pho81, in addition to stabilizing the association of Pho81 with the cyclin-dependent kinase complex. The reason why mutant Pho81 instability was not as obvious in the presence of P_i_ (Fig. 5A, lane 2) could be due to residual binding of 5-PP-IP_5_ and higher 5-PP-IP_5_ availability. In support of this, we were unable to detect WT Pho81 in a 5-PP-IP_5_-deficient background.

Our model in Fig. 6 also supports a role for 5-PP-IP_5_-Pho81 interaction in stabilizing the association of Pho81 with Pcl6/7-Pho85 to fine-tune glycogen metabolism. Although *PCL6/7* is a phosphate-responsive gene, we showed that it is dispensable for PHO pathway activation. In S. cerevisiae, Pho85 interacts with 10 cyclins, including Pho80, Plc6, and Pcl7, to regulate the PHO pathway, cell cycle, polarity, and glycogen metabolism (42–45). In addition to Pho80 and Pcl6/7, C. neoformans has five other cyclins. However, since we did not detect their association with Pho81, they are unlikely to direct phosphate-dependent activity of Pho85.

In support of our data showing that 5-PP-IP_5_ functions as an intermolecular stabilizer, there are other examples of where IP and PP-IP interactions with SPX and non-SPX domains stabilize multiprotein complexes. In a model plant Arabidopsis thaliana, 1,5-PP-IP_5_ (IP_8_) facilitates interaction of SPX1 with the PHR1 transcriptional regulator of the phosphate starvation response when phosphate is present (17). This response is triggered by a drop in the abundance of IP_8_ upon phosphate deprivation. In mammalian cells, IP_4_ stabilizes the histone deacetylase HDAC3-SMRT corepressor complex via non-SPX domain interactions to regulate gene expression. In this context, IP_4_ acts as “intermolecular glue” by wedging into a positively charged pocket formed at the interface between the two proteins (46–48).

Wild et al. (16) proposed that inositol polyphosphates communicate cytosolic phosphate levels to SPX domains to regulate phosphate uptake, transport, and storage in fungi, plants, and animals. However, our findings indicate that although 5-PP-IP_5_ interaction with the Pho81 SPX domain is essential for PHO pathway activation in C. neoformans, PHO pathway activation is not triggered by 5-PP-IP_5_ but rather by additional signaling component(s). The following evidence supports this conclusion: several reports, including this study, show that the intracellular concentration of inositol pyrophosphates, including 5-PP-IP_5_, decreases during phosphate starvation (16, 17, 36). The decreased abundance of 5-PP-IP_5_ is unlikely to trigger PHO pathway activation as the pathway is constitutively repressed in the 5-PP-IP_5_-deficient *kcs1*Δ mutant. Furthermore, Pho81-Pho85-Pho80/5-PP-IP_5_ complexes are present even when phosphate is available, and their abundance increases upon phosphate deprivation. It is likely that 5-PP-IP_5_ molecules wedged inside the complexes are partially protected from degradation and therefore have a slower turnover than free 5-PP-IP_5_. Taken together, our data suggest that preformed CKI-CDK/5-PP-IP_5_ complexes await signals other than fluctuating 5-PP-IP_5_ levels to trigger a phosphate starvation response.

Crystallographic, biochemical, and genetic analysis are required to map regions in cryptococcal Pho80 that interact with Pho81 and potentially with 5-PP-IP_5_. In S. cerevisiae, two sites on Pho80 involved in binding Pho4 and Pho81 were identified that are markedly distant to each other and the active site (45). These regions will serve as a guide to map the corresponding regions in cryptococcal Pho80. Pho81 in S. cerevisiae was also shown to inhibit Pho80-Pho85 via a novel 80-residue motif adjacent to the ankyrin repeats (called the minimal domain [MD]). This MD was shown to be necessary and sufficient for Pho81 function as a Pho85 inhibitor. This is in contrast to mammalian CKIs, which exert their regulatory function via ankyrin repeats. Domain mapping and structural studies will allow assessment of whether an MD exists in cryptococcal Pho81 to provide a second point of contact between 5-PP-IP_5_-Pho81 and cyclins.

SPX domains have been reported to undergo a conformational change upon ligand binding (16). Structural comparison of 5-PP-IP_5_-bound and free cryptococcal Pho81 may therefore shed light on whether 5-PP-IP_5_ binding induces a conformational change in Pho81. Complementary data can be obtained by creating Pho81 deletion variants to map regions required for binding 5-PP-IP_5_ and cyclins. This information will promote understanding of how conformational changes triggered by 5-PP-IP_5_ binding affect Pho81 association with Pho80-Pho85 to bring about CDK inhibition and PHO pathway activation. It will also address why the outcome of 5-PP-IP_5_–SPX domain interaction leads to different responses in different yeast species and provide insight into the physiological relevance of specific IP species in PHO pathway function.

We previously demonstrated that deletion of the cryptococcal gene encoding the transcription factor, Pho4, led to constitutive repression of the PHO pathway regardless of phosphate status, reduced growth at alkaline pH, a condition that mimics phosphate starvation and hypovirulence in a mouse inhalation model. The loss of virulence in the *pho4*Δ mutant was characterized by a higher median survival time of *pho4*Δ-infected mice relative to WT-infected mice, reduced lung colonization, and the almost complete prevention of fungal dissemination to the host brain (3). In this study, we found that growth of the Pho81-SPX^AAA^ strain was also inhibited at alkaline pH. However, Pho81-SPX^AAA^ virulence was reduced even more substantially: in contrast to infection with the *pho4*Δ mutant where only 50% of the mice succumbed to infection, all mice infected with the Pho81-SPX^AAA^ strain survived and infection burdens in lung and brain were drastically reduced. The infection kinetics and organ burdens observed for Pho81-SPX^AAA^-infected mice were similar to those observed for *pho81*Δ-infected mice, suggesting that Pho81 promotes invasive fungal disease predominantly via its association with PP-IP_5_. A potential explanation for why the Pho81 mutants are more attenuated in virulence than the *pho4Δ* mutant is that 5-PP-IP_5_-bound Pho81 regulates more than one CDK complex (see model in Fig. 6). 5-PP-IP_5_ may therefore regulate cellular functions other than phosphate homeostasis, namely, glycogen metabolism. Alternatively, Pho81 may have PP-IP_5_-dependent cellular function involving interactions with proteins other than CDK components.

In summary, we provide additional evidence of evolutionary divergence in PHO pathway regulation in a fungal pathogen of medical significance by demonstrating that interaction of the IP_7_ isomer 5-PP-IP_5_, not 1-PP-IP_5,_ with the Pho81 SPX domain is essential for PHO pathway activation. The critical roles of 5-PP-IP_5_ and Pho81 in fungal virulence are conveyed primarily via the interaction of 5-PP-IP_5_ with the Pho81 SPX domain. Finally, we demonstrate that 5-PP-IP_5_ functions as intermolecular “glue” to stabilize Pho81 association with Pho85/Pho80, providing novel mechanistic insight into how inositol pyrophosphates regulate the PHO pathway. Since Pho81 has no homologue in mammalian cells, disrupting fungal Pho81 function is a potential antifungal strategy.

## MATERIALS AND METHODS

### Fungal strains and growth conditions.

Wild-type C. neoformans var. *grubii* strain H99 (serotype A, *MAT*α) and S. cerevisiae WT strain BY4741 were used in this study. All mutant and fluorescent strains created or procured in this study are listed in Table 1 and details of their construction are provided in Materials and Methods and in the supplemental material. Routinely, fungal strains were grown in YPD (1% yeast extract, 2% peptone, and 2% dextrose). Phosphate-deficient minimal medium MM-KCl (29 mM KCl, 15 mM glucose, 10 mM MgSO_4_⋅7H_2_O, 13 mM glycine, 3.0 μM thiamine) was used to induce acid phosphatase activity. KCl was substituted with 29 mM β-glycerol phosphate for drop dilution assay media or 29 mM KH_2_PO_4_ for MM-KH_2_PO_4_. The latter was used as a control medium in which acid phosphatase activity was suppressed. In some of the experiments, the cells were grown in phosphate-depleted (low-phosphate) YPD (LP-YPD) to induce PHO pathway activation. LP-YPD was prepared as follows: 5 g yeast extract, 10 g peptone, and 1.23 g MgSO_4_ were dissolved in 475 ml of water with prolonged stirring (at least 15 min). Then, 4 ml of concentrated NH_4_OH was added dropwise, while the medium was vigorously stirred. The salts were allowed to precipitate for at least 30 min at room temperature. The medium was filtered through a 45-μm filter, supplemented with 10 g dextrose, and adjusted to pH ∼6.5 with concentrated HCl. The resulting medium was filter sterilized.

### Mice.

The Australian Resource Centre (Western Australia) provided mice (C57BL/6) for the virulence experiments. The mice weighed between 20 and 22 g (6 to 8 weeks old), and the sex was female. Maintenance and care conditions were as follows. Access to food (autoclavable rat and mouse chow supplied by Specialty Feeds) and water was unrestricted, and the light-dark cycle was 12 h. Before experiments, the acclimatization period for the animals was 1 week. Animal experiments were performed in accordance with protocol 4254.03.16, approved by the Western Sydney Local Health District animal ethics committee.

### Virulence studies in mice.

Female C57BL/6 mice (10 per infection group) were anesthetized by inhalation of 3% isoflurane in oxygen and infected with 2 × 10^5^ fungal cells via the nasal passages as described previously (4). Mice were monitored daily and euthanized by CO_2_ asphyxiation when they had lost 20% of their preinfection weight or prior if showing debilitating symptoms of infection, i.e., loss of appetite, moribund appearance, or labored breathing. Median survival differences were estimated using a Kaplan-Meier method. Posteuthanasia, the lungs and brain were removed, weighed, and mechanically disrupted in 2 ml of sterile PBS using a BeadBug (Benchmark Scientific). Organ homogenates were serially diluted and plated onto Sabouraud dextrose agar plates. Plates were incubated at 30°C for 2 days. Colony counts were performed and adjusted to reflect the total number of CFU per gram of tissue.

### Strain creation.

**(i) Pho81 SPX mutant with or without GFP tag.** Lysine residues in the Pho81 SPX domain putatively involved in binding 5-PP-IP_5_ (K^221,223,228^) were identified by sequence alignment. The Pho81 SPX mutant strain (Pho81SPX^AAA^) and its control strain (Pho81SPX) were then created in a multistep process (see Fig. S3). First, the SPX domain of *PHO81* was deleted in the WT H99 strain. Second, genomic DNA encoding the 5′ end of *PHO81*, including the SPX domain, was amplified to generate native and mutated versions. In the mutated version, codons encoding lysine 221, 223, and 228 were exchanged for those encoding alanine by overlap PCR. Native (NAT) and mutant (AAA) fragments were then fused to the *GDE2* promoter (*GDE2*p) and a dominant resistance marker by overlap PCR and used to reconstitute the *spxΔ* genotype by homologous recombination. The *GDE2* promoter (*GDE2*p) was used to replace the native *GDE1* promoter of Pho81, because Pho81 shares its promoter with the adjacent gene, CNAG_02542. *GDE2p* was a suitable choice because *PHO81/GDE1* and *GDE2* are induced to a similar extent by Pho4 during phosphate deprivation (3). In a third step, WT and mutant *PHO81* were tagged with GFP at the C terminus. The KUTAP vector containing GFP optimized for fluorescence in C. neoformans was a gift from Peter Williamson (NIAID, NIH, Bethesda, MD). Each step, including the dominant resistance markers used, is described in more detail below and is summarized in Fig. S3A.

*Step 1: deletion of the PHO81 SPX domain*. To delete the *PHO81* SPX domain (see Fig. S3A, step 1), the SPX deletion construct was created by overlap PCR, joining the 5′ flank, the hygromycin resistance cassette with the *ACT1* promoter and *GAL7* terminator (Hyg^r^), and the 3′ flank. The 5′ flank, consisting of 977 bp upstream of the *PHO81* gene, was PCR amplified from genomic DNA using the primers PHO81_ots_s and (HygB)PHO81-5′a. The 3′ flank, consisting of 1,275 bp downstream of the SPX domain, was PCR amplified using the primers (HygB)PHO81-3′s and PHO81_ots_3′a. Hyg^r^ was PCR amplified with the primers Neo-s and HygB_a (49). The three fragments were fused together using the primers PHO81_5′s and PHO81_3′flank-a, and the resulting 4,955-bp product was used to delete the SPX domain from *PHO81* in the H99 WT strain, using biolistic transformation (50). Hygromycin B-resistant (Hyg^r^) colonies were screened by PCR amplification across the SPX external recombination junctions using the primers indicated in Table S1 in the supplemental material. A successful transformant was used in step 2 to create the Pho81SPX and Pho81SPX^AAA^ strains.

*Step 2: reconstitution of spxΔ with SPX (Pho81SPX) or SPX^AAA^ (Pho81SPX^AAA^)*. For the reconstitution of *spxΔ* with SPX (Pho81SPX) or SPX^AAA^ (Pho81SPX^AAA^) (see Fig. S3A, step 2), the following three fragments were fused together by overlap PCR: (i) the neomycin resistance cassette with *ACT1* promoter and *TRP1* terminator (Neo^r^), (ii) the *GDE2p* to drive expression of *PHO81*, and (iii) the *PHO81* gene sequence consisting of the 1,070-bp SPX domain (native or AAA) and 1,275 bp downstream of SPX. Neo^r^ was PCR amplified from pJAF1 using the primer pair Neo-s and Neo-a. H99-derived *GDE2*p was PCR amplified using the primer pair (NEO)GDE2p-s and (SPX)GDE2p-a. The 2,345-bp native *PHO81* SPX fragment (SPX^Nat^) was PCR amplified using the primer pair SPX-start-s and PHO81_ots_3′a. The mutant *PHO81* SPX fragment (SPX^AAA^) was created by PCR amplifying the 1,070-bp SPX domain and 1,275 bp downstream of SPX using the primer pairs SPX-start-s/Pho81-AAA-a and Pho81-AAA-s/PHO81_ots_3′a, which introduced the mutation at the adjoining ends. The two fragments were then fused together by overlap PCR, using the primer pairs SPX-start-s and PHO81_ots_3′a, to introduce the A^221,223,228^ mutations in the overlapping region. A third PCR was then used to fuse Neo^r^-*GDE2p*-SPX^Nat^ or Neo^r^-*GDE2p*-SPX^AAA^ using the primer pair Neo-s and PHO81_3′flank-a. The final products were introduced into the *Δspx* strain created in step 1, resulting in strains Pho81SPX^Nat^ and Pho81SPX^AAA^. Geneticin-resistant, hygromycin-sensitive transformants were screened by PCR amplification across the NeoR-GDE2p-SPX recombination junctions (see Fig. S3B) using the primers indicated in Table S1.

*Step 3: GFP-tagging Pho81SPX and Pho81SPX^AAA^*. For GFP-tagging Pho81SPX and Pho81SPX^AAA^ (see Fig. S1B, step 3), a construct consisting of (i) the 5′ flank, encoding 865 bp of the 3′ end of the *PHO81* gene without the stop codon; (ii) GFP fused to the nourseothricin resistance cassette (Nat^r^); and (iii) the 3′ flank, encoding 866 bp downstream of the *PHO81* gene, was created by overlap PCR. The 5′ flank was PCR amplified from H99 genomic DNA using the primer pair Pho81-ots-s and Pho81-3f-a (GFP). Using the primers GFP-start-s and Neo-a, GFP-Nat^r^ (3,116 bp) was PCR amplified from the pCR21 vector (Invitrogen) into which GFP-Nat^r^ had previously been cloned. The 3′ flank was PCR amplified from genomic DNA using the primer pair Pho81-3f-s_(NEO) and Pho81-ots-a. These three overlapping fragments were fused by a final overlap PCR using the primer pair Pho81-5f-s and Pho81-3f-a. The final product was introduced into strains *Pho81SPX* and *Pho81SPX^AAA^*, creating *GDE2p*-Pho81-GFP and *GDE2p*-Pho81^AAA^-GFP, respectively, using biolistic transformation. Nourseothricin-resistant transformants were screened by PCR amplifying regions across recombination junctions (see Fig. S3B) using the primers described in Table S1.

**(ii) PHO81 deletion and rescue.** A *PHO81* gene deletion construct was created by joining the 5′ flank (963 bp of genomic DNA upstream of the *PHO81* coding sequence), the hygromycin B resistance (Hyg^r^) cassette (with the *ACT1* promoter and *GAL7* terminator), and the 3′ flank (1,424 bp of genomic DNA downstream of the *PHO81* coding sequence). The three fragments were fused by overlap PCR using the primer pair PHO81_5′s and PHO81_3′a. This deletion construct was used to transform the H99 WT strain using biolistics (50), creating *Δpho81:HYGB*. Hygromycin-resistant colonies were screened by PCR amplification across the 5′ and 3′ recombination junctions using the primers indicated in Fig. S4 and Table S1 to confirm that homologous recombination had occurred at the correct site.

To create the *PHO81* reconstituted strain (*Δpho81*+*PHO81*), the genomic *PHO81* locus, which comprised the coding region and 5,727 bp upstream and 345 bp downstream of the coding region, was PCR amplified from genomic DNA prepared from the H99 WT strain using the primer pair PHO81_5′s and (NEO)PHO81-Rec-5′a. The neomycin resistance (Neo^r^) cassette (with the *ACT1* promoter and *TRP1* terminator) was PCR amplified from pJAF (51) using the primer pair Neo-s and Neo-a. The two fragments were fused by overlap PCR using the primer pair PHO81-Rec-5′s and Neo-a, and the resulting gene fusion was used to transform the *Δpho81* mutant using biolistics as described above. Neomycin-resistant transformants were screened for their ability to secrete acid phosphatase (Aph1) using the colorimetric pNPP reporter assay described previously (3). This phenotype was lost following deletion of *PHO81* in the WT strain. Transformants that tested positive for secreted acid phosphatase activity were tested further for the presence of the *PHO81* gene by PCR amplifying an internal region of the *PHO81* locus from genomic DNA using the primers indicated in Fig. S4 and Table S1.

**(iii) PHO85-mCherry strain.** To create a WT C. neoformans strain expressing *PHO85* as an mCherry fusion protein, a construct consisting of (i) the 5′ flank, 1,141 bp of 3′ end of the *PHO85* gene without the stop codon; (ii) mCherry; (iii) the hygromycin resistance cassette (Hyg^r^) with the *ACT1* promoter and *GAL7* terminator; and (iv) the 3′ flank, 988 bp downstream of the *PHO8*5 gene, was created by overlap PCR. The 5′ flank was PCR amplified from H99 WT genomic DNA using the primer pair PHO85-int-s1 and (mCherry)PHO85-a. The mCherry was PCR amplified from pNEO-mCherry vector using the primer pair (PHO85)mCherry-s and (ActP)-mCherry-a. Hyg^r^ was generated using the primer pair Neo-s and HygB-a. The 3′ flank was PCR amplified from H99 WT genomic DNA using the primer pair (Gal7t)PHO85-3′flank-s and PHO85-3′flank-a1. These four fragments were fused by overlap PCR using the primer pair PHO85-int-s3 and PHO85-3′flank-a3, and the final product was then used to transform H99 WT using biolistic transformation (50). Hygromycin-resistant colonies were screened by PCR amplification across the recombination junctions using primers listed in Table S1.

**(iv) Pho81SPX-GFP in a WT and *kcs1Δ* background.** A DNA construct consisting of (i) the 5′ flank, encoding 865 bp of the 3′ end of the *PHO81* coding region minus the stop codon, (ii) GFP fused to the nourseothricin resistance cassette (Nat^r^), and (iii) the 3′ flank, encoding 866 bp downstream of the *PHO81* coding region, was amplified by PCR from genomic DNA prepared from the Pho81SPX-GFP strain used in Fig. 5 using the primer pair Pho81-5f-s and Pho81-3f-a. The final 4,559-bp product was introduced into the *kcs1Δ:NEO* strain (4) using biolistic transformation. Nourseothricin-resistant transformants were screened by PCR by amplifying regions across the recombination junctions using the primers described in Table S1.

### Assessing PHO pathway activation.

**(i) Acid phosphatase reporter assay.** Extracellular acid phosphatase (APase) activity associated with the *APH1* gene product was measured as previously described (5). Briefly, YPD overnight cultures were centrifuged, and the pellets were washed twice with water and resuspended in PHO pathway-inducing and noninducing medium (see above) at an optical density at 600 nm (OD_600_) of 1. The cultures were incubated at 30°C for 3 h or as indicated otherwise. After incubation, 200 μl of each culture was centrifuged, and the pellets were resuspended in 400 μl of APase reaction mixture (50 mM sodium acetate [pH 5.2], 2.5 mM *p*-nitrophenyl phosphate [pNPP]). Reactions were performed at 37°C for 5 to 15 min, which was the time determined to be within the linear range of APase activity (5). Reactions were stopped by adding of 800 μl of 1 M Na_2_CO_3_. APase-mediated hydrolysis of pNPP was quantified spectrophotometrically at 420 nm. Any growth difference among strains was corrected by measuring the OD_600_ prior to performing the assay, and the APase activity was calculated as OD_420_/OD_600_. In some cases, the APase activity was normalized to the WT and expressed as a fold change. In the experiment where PHO pathway activation in the absence of phosphate was measured over a 2-day time course, 10 to 300 μl of culture was used for the APase activity assay, with smaller amounts needed at longer induction times to prevent reaction saturation due to increased culture growth. All assays were performed in biological triplicate.

**(ii) Quantitative PCR.** RNA extraction, cDNA synthesis, and qPCR of PHO genes in C. neoformans strains was performed as described previously (3). The sequences of primers used for qPCR are listed in Table S1.

### Fractionating ^3^H-labeled inositol polyphosphates.

[^3^H]inositol labeling of fungal cells was performed as previously described (52), with modifications (4). Overnight fungal cultures grown in YPD were diluted to an OD_600_ of 0.05 in fresh YPD containing 10 mCi/ml [^3^H]myo-inositol (Perkin-Elmer) and incubated until an OD_600_ of >6. The cells were pelleted, washed, and resuspended in MM with or without phosphate (as indicated). After an additional 2 h of incubation, fungal cells were pelleted, washed, and snap-frozen in liquid nitrogen. To extract inositol polyphosphates, the cells were resuspended in extraction buffer (1 M HClO_4_, 3 mM EDTA, 0.1 mg/ml IP_6_) and homogenized with glass beads using a bead beater. Debris was pelleted, and the supernatants were neutralized (1 M K_2_CO_3_, 3 mM EDTA) and stored at 4°C. The radiolabeled inositol polyphosphates were fractionated by anion-exchange high-pressure liquid chromatography (HPLC).

### Creation of a 5-PP-IP_5_ affinity capture resin.

Synthesis of resin-bound 5PCP-IP_5_, a diphosphoinositol polyphosphate analog containing a nonhydrolyzable bisphosphonate group in the 5-position as described in detail by Wu et al. (37). This bisphosphonate analog closely resembles the natural molecule, both structurally and biochemically, while exhibiting increased stability toward hydrolysis in a cell lysate (53).

### Assessing binding of Pho81 to 5-PP-IP_5_.

GFP-labeled strains were grown for 5 h in LP-YPD (P_i_– culture) or overnight in YPD (P_i_+ culture). The cells were pelleted by centrifugation, and lysates were prepared as described above using lysis buffer (50 mM Tris [pH 7.5], 150 mM NaCl, 0.05% Triton X-100, 1 mM EDTA, 1 mM phenylmethylsulfonyl fluoride [PMSF], 1 μl/10 mg cell pellet fungal protease inhibitor cocktail [Sigma, catalog no. P8215]). Protein in each lysate was adjusted to ∼8 mg/ml to correct for growth differences and incubated with 5-PCP-IP_5_-conjugated resin or phosphate (P_i_)-conjugated resin as a control (∼200 μl of slurry) (37). Incubation was allowed for 2 h at 4°C. The resin was pelleted by centrifugation, and the supernatant was removed. The resin was then washed three times with lysis buffer. Resin-bound proteins were eluted by resuspension in SDS-PAGE loading buffer and resolved by SDS-PAGE. GFP-tagged proteins were detected by Western blotting with anti-GFP antibody (Santa Cruz Biotechnology, catalog no. sc-9996).

### Immunocapture of fluorescent Pho81.

Cells were grown overnight in YPD or LP-YPD as indicated. Cell pellets were collected and snap-frozen. At least 120 and 350 mg of cell pellet was used for immunoprecipitation/Western blotting and immunoprecipitation/1D-LC-MS, respectively. Cell pellets were resuspended in 2 volumes of lysis buffer (0.1% NP-40, 250 mM NaCl, 50 mM sodium fluoride, 5 mM EDTA, 50 mM Tris-HCl [pH 7.5], 1 mM DTT, 1 mM PMSF, 1 μl/10 mg cell pellet fungal protease inhibitor cocktail [Sigma, catalog no. P8215]). Lysates were prepared by bead beating the cells in the presence of glass beads (425 to 600 μm), followed by centrifugation at 4°C at maximum speed. GFP-Trap agarose (Chromotek gta-20) was used to immunoprecipitate GFP-tagged Pho81. Protein G-Sepharose 4 Fast Flow (GE Healthcare, catalog no. 17061801) and anti-mCherry antibody (ab-167453; Abcam) were used to immunoprecipitate Pho85-mCherry. Sepharose beads were washed three times in lysis buffer and incubated with the lysate for 2 to 3 h at 4°C. After lysate incubation, the Sepharose was washed three times with lysis buffer and resuspended in SDS-PAGE loading buffer, and the solubilized proteins were resolved by SDS-PAGE. Cell lysates were run as a loading control.

**(i) Western blotting.** GFP-tagged proteins were detected using anti-GFP antibody (Santa Cruz Biotechnology, sc-9996) and Pho85 was detected using anti PSTAIRE (1:200 dilution; Santa Cruz Biotechnology, sc-53) or anti-PSTAIR antibody (1:200 dilution; Merck, catalog no. 06-923), followed by Amersham ECL anti-rabbit IgG, HRP-linked F(ab′)_2_ fragment (from donkey; 1:8,000 dilution). Anti-PSTAIR antibodies also detect cryptococcal Cdc2 (Cdc28 in S. cerevisiae) in total cell lysates since both Cdc2 and Pho85 contain PSTAIRE motif. Cdc2 was therefore used as a control for protein loading (54). Similar levels of Cdc2 (the main cyclin-dependent kinase in yeast) were detected in whole lysates, confirming that each immunoprecipitation had been performed from lysates containing similar levels of total protein.

**(ii) 1D-LC-MS.** SDS gels were stained using a colloidal blue staining kit (LC6025; Life Technologies) according to the manufacturer’s protocol.

Each lane was cut into five equal pieces and the proteins within each piece were trypsin treated and analyzed by mass spectrometry. Briefly, the gel slices were diced up and destained in a 60:40 solution of 40 mM NH_4_HCO_3_ (pH 7.8)–100% acetonitrile for 1 h. Gel pieces were vacuum-dried and then rehydrated with a 12-ng/μl trypsin (Promega) solution at 4°C for 1 h. Excess trypsin was removed, and gel pieces were covered with 40 mM NH_4_HCO_3_ and then incubated overnight at 37°C. Peptides were concentrated and desalted using C18 Zip-Tips (Millipore, Bedford, MA) according to the manufacturer’s instructions. Peptides were resuspended in 10 μl of 3% (vol/vol) acetonitrile–0.1% (vol/vol) formic acid and briefly sonicated. Samples were separated by nano-LC using an Ultimate 3000 HPLC and autosampler system (Thermo Fisher Scientific, Scoresby, UK) coupled to an in-house fritless nano-LC 75 μm × 40 cm column packed with ReproSil Pur 120 C_18_ in the stationary phase (1.9 μm, Dr. Maisch GmbH, Ammerbuch, Germany). LC mobile phase buffers were comprised of solvent A (0.1% [vol/vol] formic acid) and solvent B (80% [vol/vol] acetonitrile, 0.1% [vol/vol] formic acid). Peptides were eluted using a linear gradient of 5% B to 35% B over 60 min, followed by a 95% B wash over 1 min at a flow rate of 250 nl/min. The LC was coupled to a Q Exactive Plus Orbitrap mass spectrometer (Thermo Fisher Scientific). The column voltage was 2,300 V, and the heated capillary was set to 275°C. Positive ions were generated by electrospray, and the Orbitrap was operated in data-dependent acquisition mode. A survey scan of 350 to 1,550 *m/z* was acquired (resolution = 35,000, with an accumulation target value of 3,000,000 ions) with lockmass enabled. Up to 20 of the most abundant ions (>1.7E5 ions), with charge states of ≥+2 and <+6, were sequentially isolated and fragmented, and a target value of 100,000 ions were collected. Ions selected for tandem mass spectrometry (MS/MS) were dynamically excluded for 20 s. The data were analyzed using Proteome Discoverer v2.3 (Thermo Fisher Scientific) and Mascot v2.4 (Matrix Science, London, UK). The search parameters included the following variable modifications: oxidized methionine, acetyl (protein N-term), deamidated asparagine/glutamine, and carbamidomethyl cysteine. The enzyme was set to trypsin, and the precursor mass tolerance was set to 10 ppm, while the fragment tolerance was 0.05 Da. The databases were the C. neoformans H99 from FungiDB v44 (fungidb.org) and a common contaminants database. Proteins were quantified by using the minora feature detector node and precursor ion quantifier node.

### Statistics.

Statistical analysis for virulence studies was performed using SPSS (SPSS, Chicago, IL), SAS Studio (SAS Institute, Inc.), and Prism (v8.0; GraphPad Software, Inc., San Diego, CA) statistical software. Differences in mouse mortality were determined by comparing survival curves of different infected groups using a log-rank test. Differences in CFU were assessed by using a two-sample *t* test or a Mann-Whitney U test when data were not normally distributed. One-way analysis of variance (ANOVA) or a Student *t* test was used in all other experiments, as indicated in the figure legends. All plotted data represent means ± the standard deviations. Results were considered significant at a *P* value of <0.05.
