# Supplementary figures and images for: IP7-SPX Domain Interaction Controls Fungal Virulence by Stabilizing Phosphate Signaling Machinery
Source: mBio. 2020 Oct 20;11(5):e01920-20. doi: 10.1128/mBio.01920-20 (PMC7587432; doi:10.1128/mBio.01920-20)

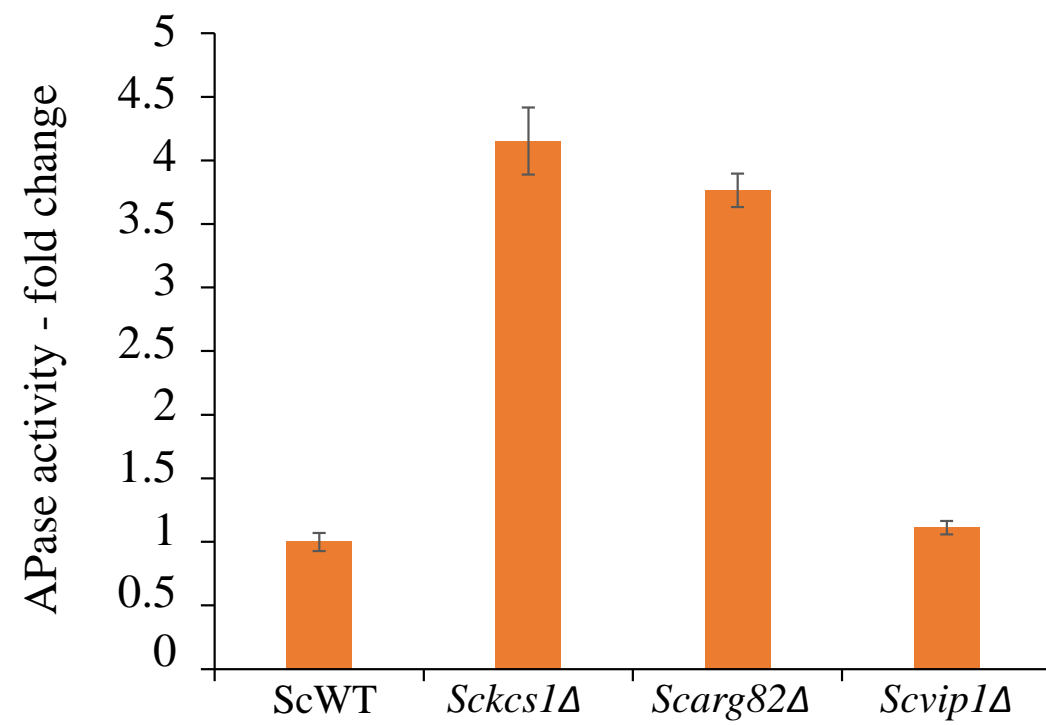

**Figure S1**

Supplement: FIG S1 [file mBio.01920-20-sf001.pdf]

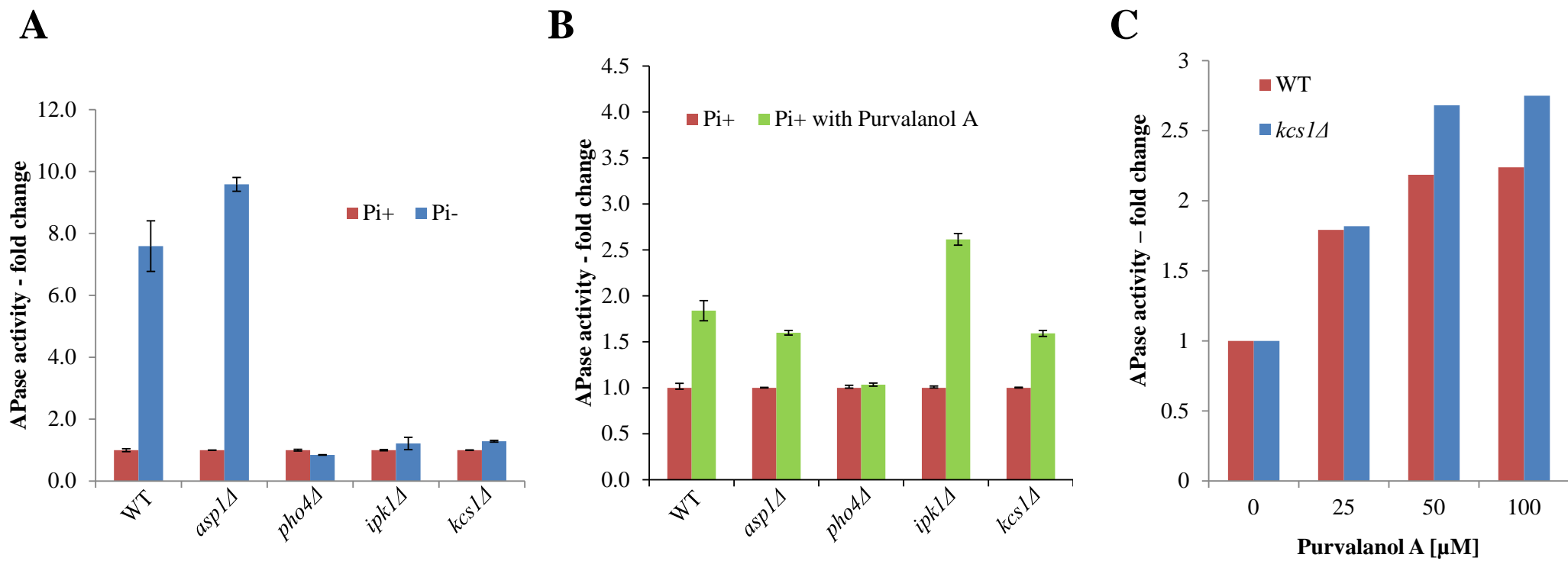

**Figure S2**

Supplement: FIG S2 [file mBio.01920-20-sf002.pdf]

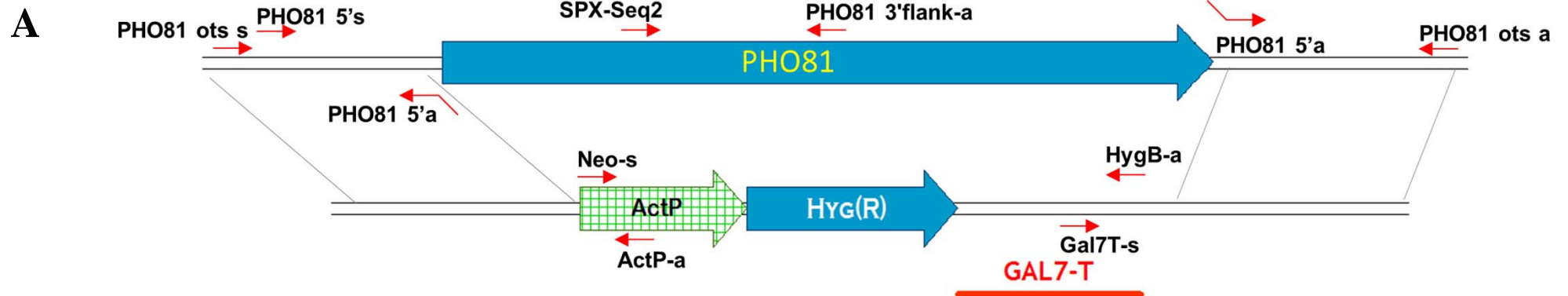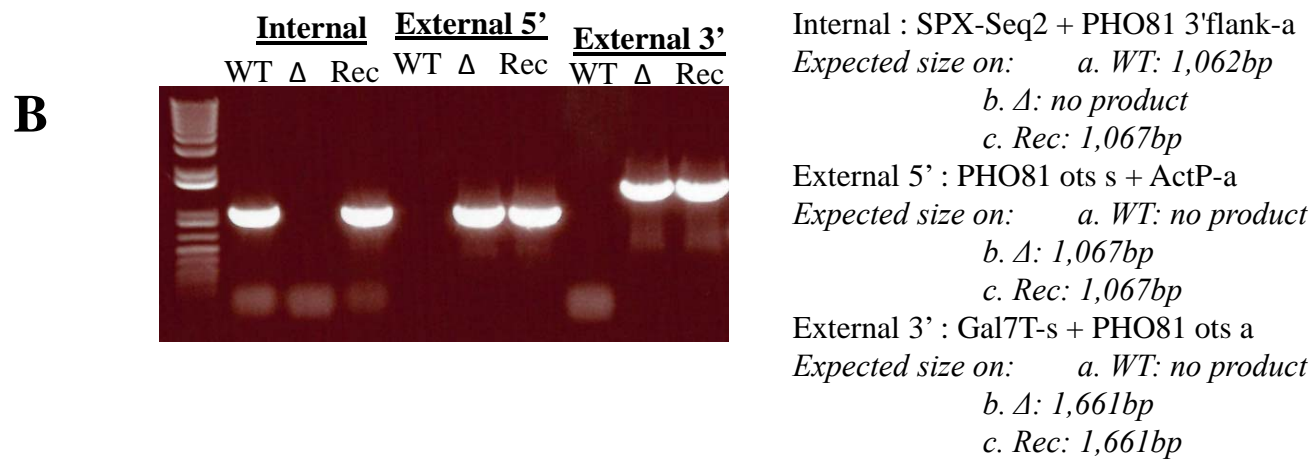

**Figure S4**

Supplement: FIG S4 [file mBio.01920-20-sf004.pdf]

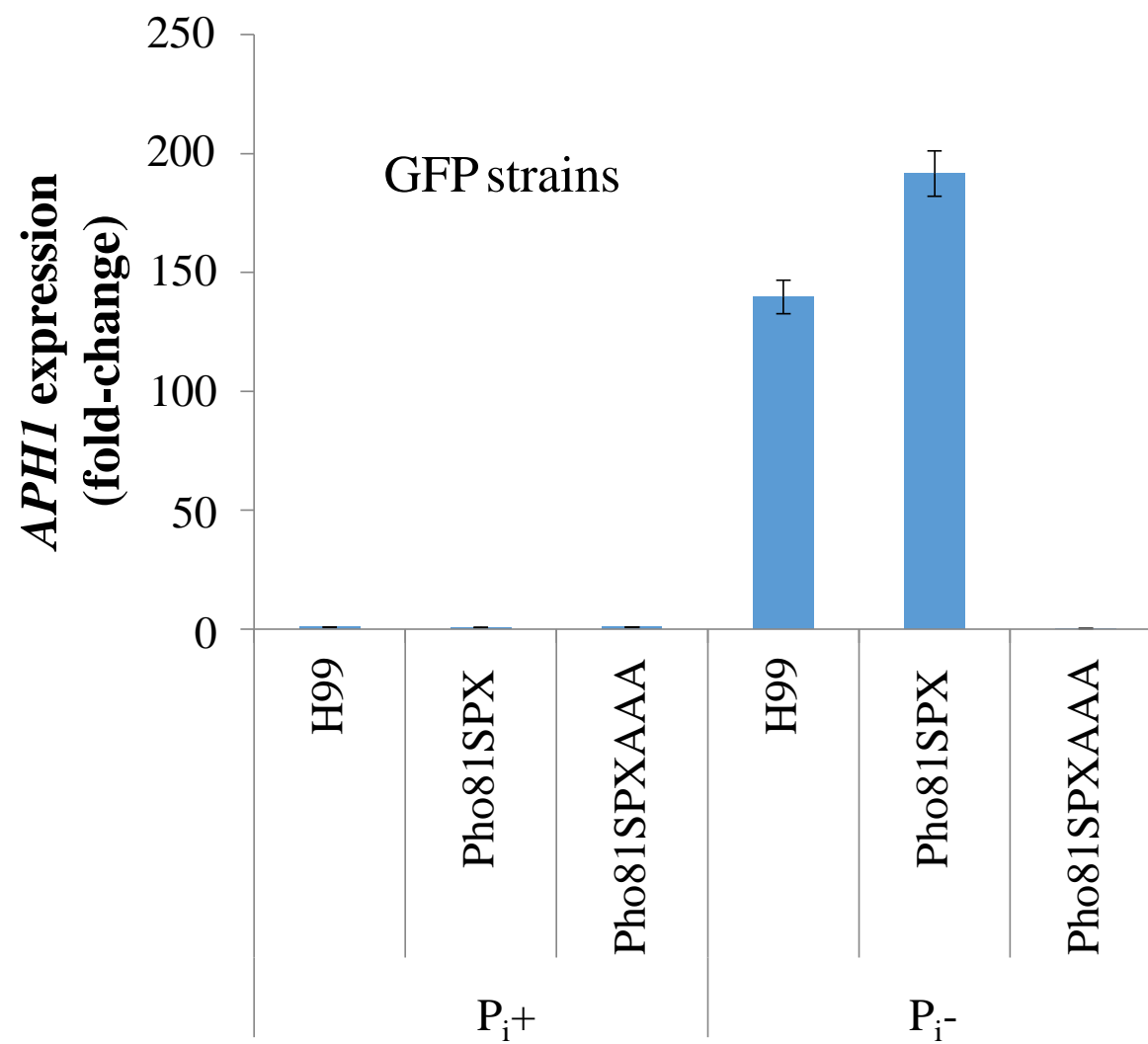

**Figure S5**

Supplement: FIG S5 [file mBio.01920-20-sf005.pdf]

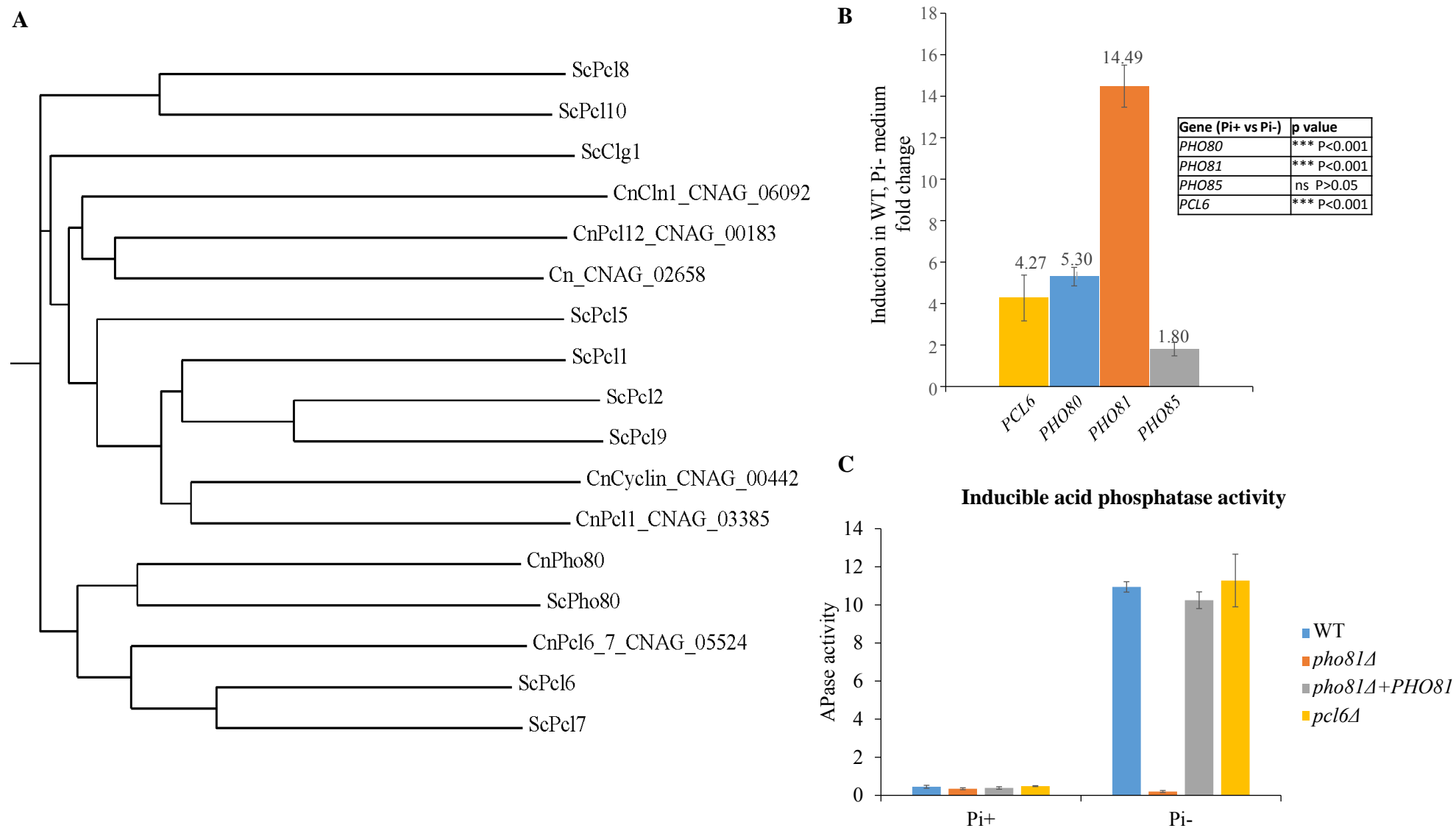

**Figure S6**

Supplement: FIG S6 [file mBio.01920-20-sf006.pdf]

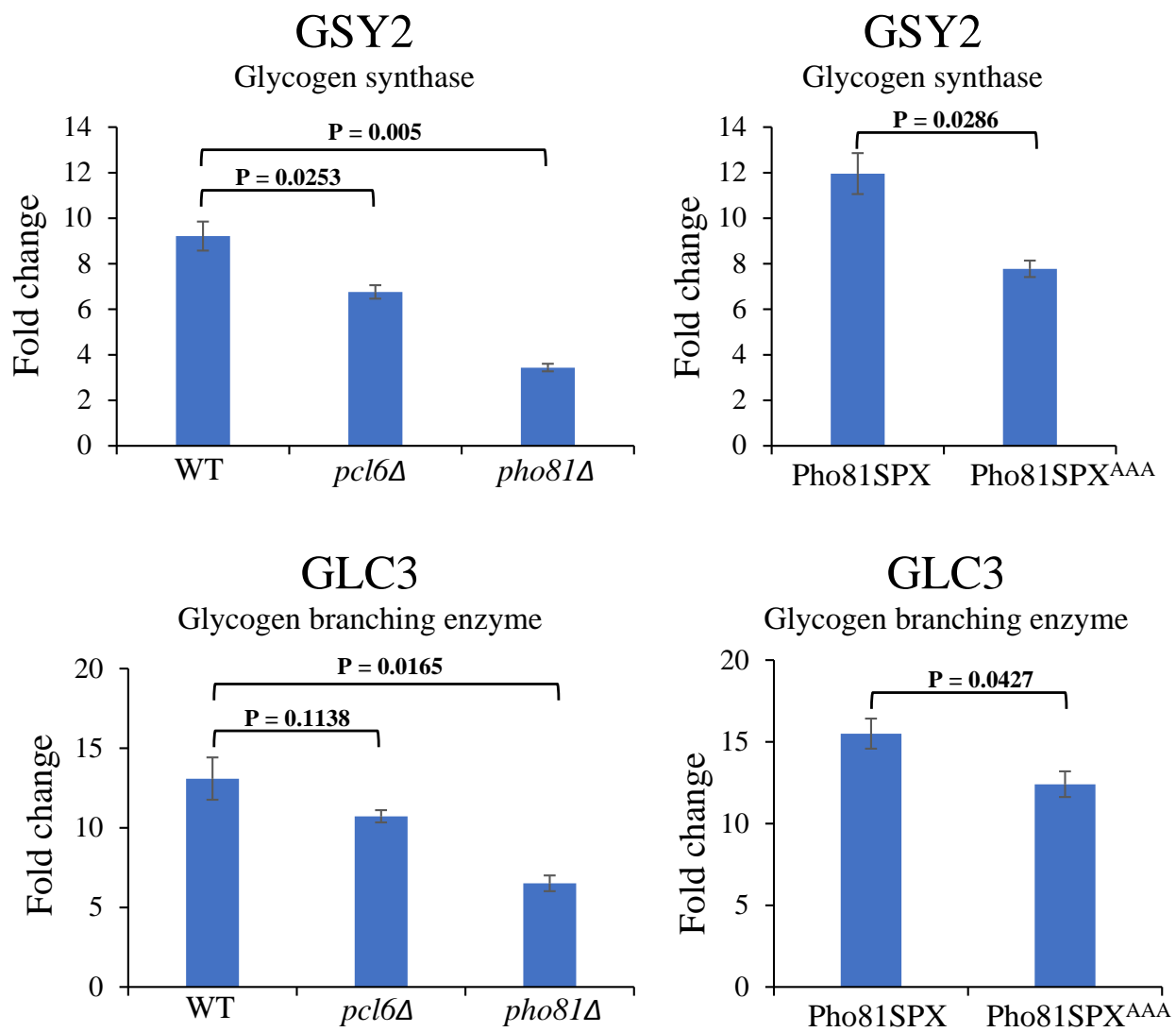

**Figure S7**

Supplement: FIG S7 [file mBio.01920-20-sf007.pdf]

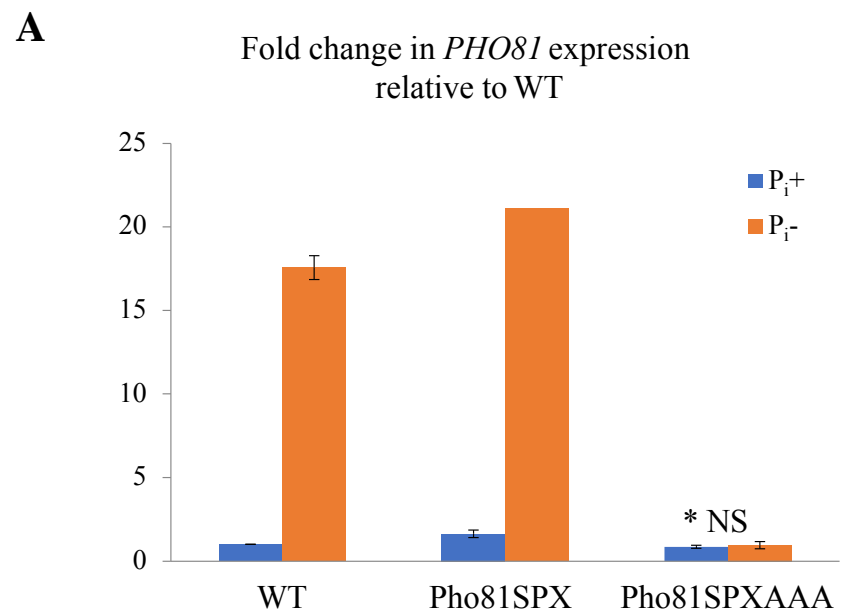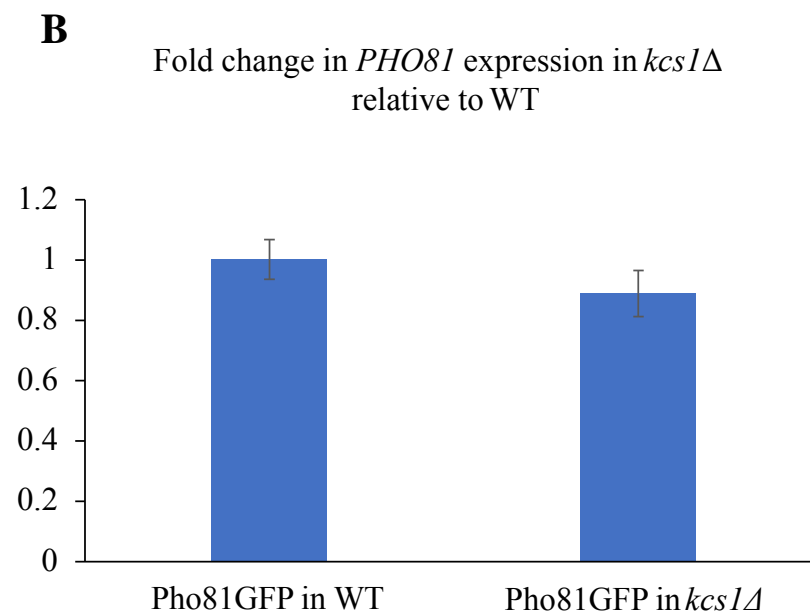

**Figure S8**

Supplement: FIG S8 [file mBio.01920-20-sf008.pdf]
