# Supplementary material for: IP7-SPX Domain Interaction Controls Fungal Virulence by Stabilizing Phosphate Signaling Machinery
Source: mBio. 2020 Oct 20;11(5):e01920-20. doi: 10.1128/mBio.01920-20 (PMC7587432; doi:10.1128/mBio.01920-20)
Supplement: FIG S3 [file mBio.01920-20-sf003.pdf]

A

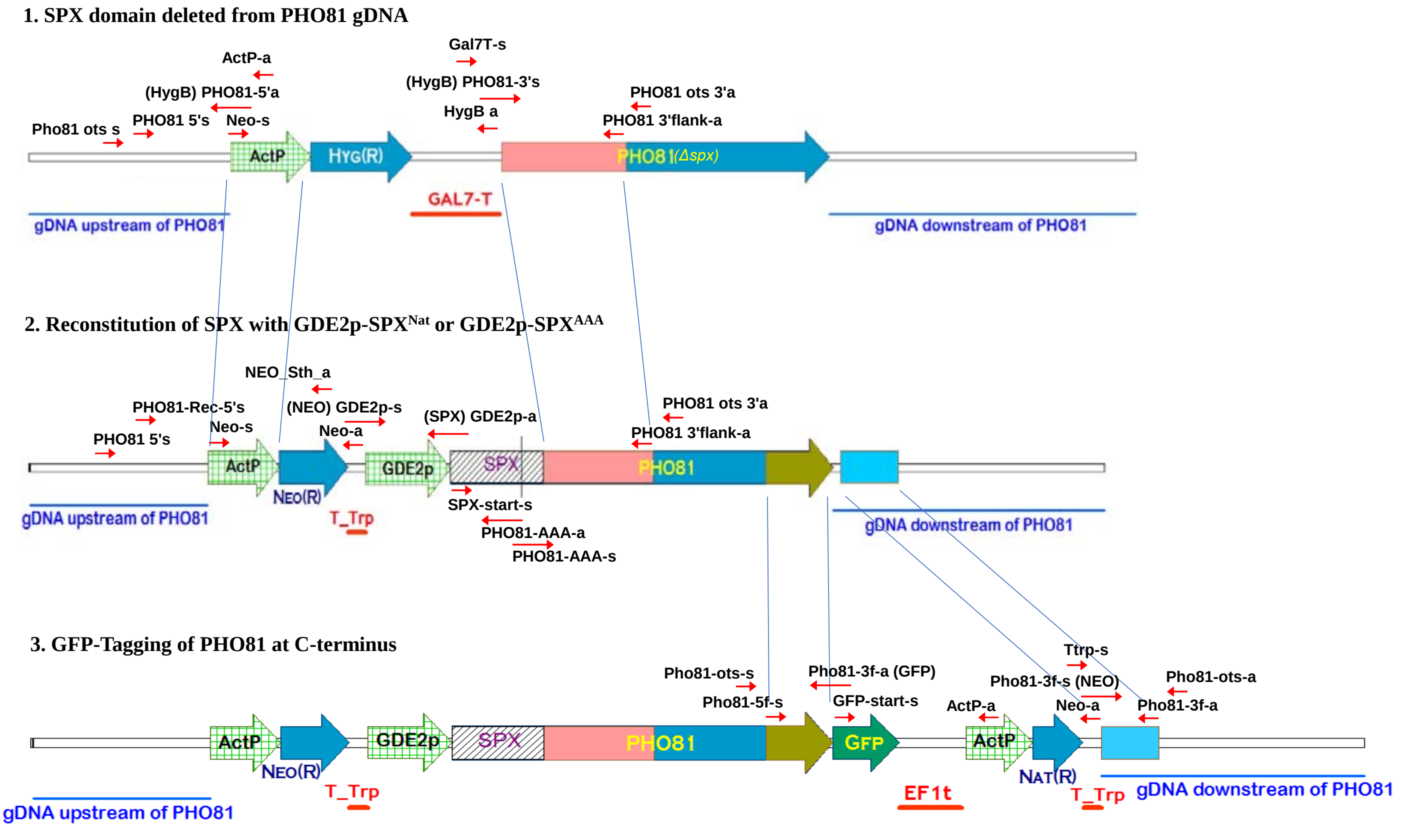

B

1. Verification PCR of SPX deletion

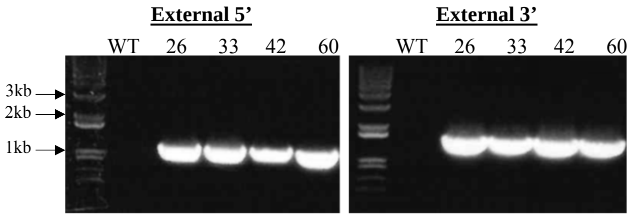

Expect lack of product in WT for both PCRs
Expected size for the correct clones on:

- external 5' = 1,102bp
- external 3' = 1,534bp

All 4 clones were correct. Clone#42 used for next step

2. Verification of SPX^{Nat} and SPX^{AAA} reconstitution

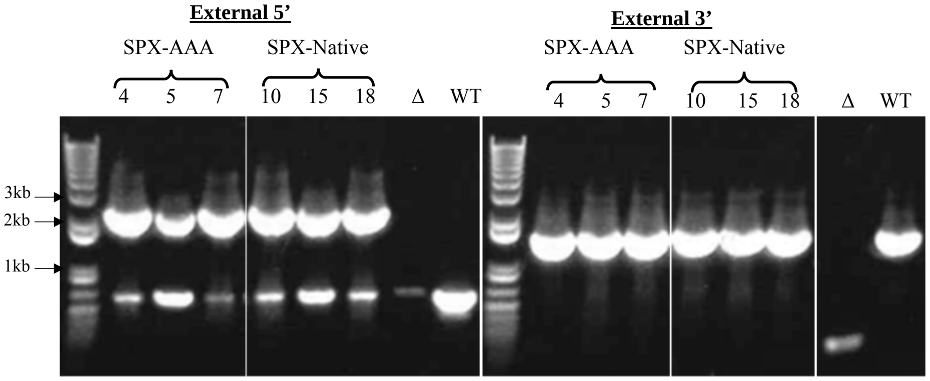

External 5' : PHO81-Rec-5's + NEO_Sth_a
Expected size on:
a. correct clone: 2,178bp (clone #4 for next step)
b. WT : no product
c. Δ (background strain) : no product

External 3' : Pho81-AAA-s + PHO81 ots 3'a
Expected size on:
a. correct clone: 1,546bp (clone #10 for next step)
b. WT : 1,546bp
c. Δ (background strain) : no product

3. Verification of GFP-tagging of Pho81SPX and Pho81SPX^{AAA}

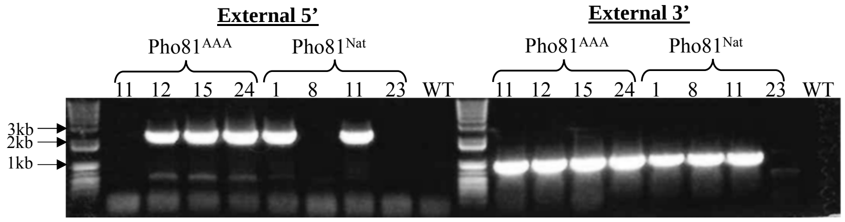

Expect lack of product in WT for both PCRs
Expected size for the correct clones on:

- external 5' = 2,464bp
- external 3' = 914bp

For Pho81^{AAA}, clones #12, #15 and #24 were correct. Clone #15 was used for all experiments
For Pho81^{Nat}, clones #1 and #11 were correct. Clone #11 was used for all experiments

Figure S3
