# Supplementary material for: IP7-SPX Domain Interaction Controls Fungal Virulence by Stabilizing Phosphate Signaling Machinery
Source: mBio. 2020 Oct 20;11(5):e01920-20. doi: 10.1128/mBio.01920-20 (PMC7587432; doi:10.1128/mBio.01920-20)
Supplement: TABLE S1 [file mBio.01920-20-st001.pdf]

Table S1. Primers used for all constructs and qPCR

Primer Name	Sequence (5' to 3')	Primer Name	Sequence (5' to 3')	Description
PHO81 ots s	ATGTATTATCCTGCTCTGTCGCTCCA	(HygB) PHO81-5'a	CTCCAGCTCACATCCTCGCAGAGCAGATGGAGAGCCTGGAGA	SPX deletion. Upstream region of PHO81 (and SPX domain)
Neo-s	CTGCGAGGATGTGAGCTGGAG	HygB a	TCTCTATACGGCGATTGGCGGA	Hygromycin resistance cassette
(HygB) PHO81-3's	TCCGCCAATCGCCGTATAGAGAGCGTAGGGAGCAATAGGAGTGT	PHO81 ots 3'a	GTATCAACTCAAACTCTTCGGCAGGA	SPX deletion. Downstream region of SPX domain
PHO81 5's	CCCATACTTGCCTTCATACCCTTCAG	PHO81 3'flank-a	TCTGAACCGGGCGACAATGCT	Overlap PCR to fuse all 3 fragments for SPX deletion
PHO81 ots s	ATGTATTATCCTGCTCTGTCGCTCCA	ActP-a	TGTTGTTACCATCATCCTCTCCTC	Verification PCR to confirm SPX deletion, external 5' recombination
Gal7T-s	CTGGTTCCTGCTGGTATTGGCTT	PHO81 ots 3'a	GTATCAACTCAAACTCTTCGGCAGGA	Verification PCR to confirm SPX deletion, external 3' recombination
Neo-s	CTGCGAGGATGTGAGCTGGAG	Neo-a	GGAGCCATGAAGATCCTGAGG	Neomycin resistance cassette
(NEO) GDE2p-s	CCTCAGGATCTTCATGGCTCCGCTGCCCTTCTATTTCATTTGTTATTGC	(SPX) GDE2p-a	ACGTAAGGCTGTACCTTCATCTTGTCGTAATTATTACTGA	GDE2p amplification
SPX-start-s	ATGAAGGTACAGCCTTACGT	PHO81 ots 3'a	GTATCAACTCAAACTCTTCGGCAGGA	Amplification of SPX^{Nat} and overlap PCR to fuse SPX^{AAA} fragment 1 and fragment 2
SPX-start-s	ATGAAGGTACAGCCTTACGT	Pho81-AAA-a	TGCATCCCATTTTGCCAAGATTGCACGGAAGCCTGTAGCGTTGATTTC	SPX^{AAA} fragment 1 (see Figure S2A, step 2)
Pho81-AAA-s	GCAATCTTGGCAAAATGGGATGCACGGAGTAAGAGTAATACCAAGGAACTC	PHO81 ots 3'a	GTATCAACTCAAACTCTTCGGCAGGA	SPX^{AAA} fragment 2 (see Figure S2A, step 2)
Neo-s	CTGCGAGGATGTGAGCTGGAG	PHO81 3'flank-a	TCTGAACCGGGCGACAATGCT	Overlap PCR to fuse all fragments for reconstitution with either GDE2p-SPX^{Nat} or GDE2p-SPX^{AAA}
PHO81-Rec-5's	ATGTTTGCGATGGGCTGTGTGA	NEO_Sth_a	CGATAGAAGGCGATGCGCTG	Verification PCR to confirm SPX reconstitution, external 5' recombination
Pho81-AAA-s	GCAATCTTGGCAAAATGGGATGCACGGAGTAAGAGTAATACCAAGGAACTC	PHO81 ots 3'a	GTATCAACTCAAACTCTTCGGCAGGA	Verification PCR to confirm SPX reconstitution, external 3' recombination
GFP-start-s	TCCAAGGGTGAGGAGCTCTTC	Neo-a	GGAGCCATGAAGATCCTGAGG	Amplification of GFP-Nat from plasmid
Pho81-ots-s	AAGGTAGGAAGGGAGAGCGGT	Pho81-3f-a (GFP)	GAAGAGCTCCTCACCCTTGGAAATAGTAAGAGTCATAATCCTATCACC	GFP-Tagging of PHO81. Amplification of 3'end of PHO81 gene (5' flank)
Pho81-3f-s (NEO)	CCTCAGGATCTTCATGGCTCCGGCTTCTGTATCTTTGGCTGAAA	Pho81-ots-a	GAGGACGAAGAGGAAGATGACAG	GFP-Tagging of PHO81. Amplification of downstream region of PHO81 gene (3' flank)
Pho81-5f-s	AGGTAGGAAAGAAAGTTGGTGG	Pho81-3f-a	GCTCTCCATTACGCCCTTTCA	Overlap PCR to fuse all 3 fragments to generate PHO81-GFP-Nat®
Pho81-ots-s	AAGGTAGGAAGGGAGAGCGGT	ActP-a	TGTTGTTACCATCATCCTCTCCTC	Verification PCR to confirm integration of GFP tag at the C-terminus of PHO81, external 5' recombination
Ttrp-s	CTACAGACAACAATACCATCCTTCC	Pho81-ots-a	GAGGACGAAGAGGAAGATGACAG	Verification PCR to confirm integration of GFP tag at the C-terminus of PHO81, external 3' recombination
PHO85-int-s1	TCCTTTAGATTCGCAGAGATGATTACAG	(mCherry)PHO85-a	ATCCTCCTCGCCCTTGCTCACATGGTGATGCCCATGCTGTC	mCherry-tagging of PHO85. Amplification of 3'end of PHO85 gene (5'flank)
(PHO85)mCherry-s	GACAGCATGGGCATCACCATGTGAGCAAGGGCGAGGAGGAT	(ActP)-mCherry-a	CTCCAGCTCACATCCTCGCAGGTACCAATCTATCCCTCTCT	mCherry-tagging of PHO85. Amplification of mCherry from pNEO-mCherry plasmid
Neo-s	CTGCGAGGATGTGAGCTGGAG	HygB a	TCTCTATACGGCGATTGGCGGA	mCherry-tagging of PHO85. Amplification of Hygromycin resistance cassette
(Gal7t)PHO85-3'flank-s	TCCGCCAATCGCCGTATAGAGAATACTGTTTCTTGAATCATT	PHO85-3'flank-a1	GAGGATAATGAGAGATGGCAGGCTAAA	mCherry-tagging of PHO85. Amplification of downstream region og PHO85 gene (3'flank)
PHO85-int-s3	CACACCAAGCGATGCCACCA	PHO85-3'flank-a3	CGCCACCTCTTCCTCTCTTGTT	Overlap PCR to fuse all 4 fragments to generate PHO85-mCherry-HygR
PHO85-int-s1	TCCTTTAGATTCGCAGAGATGATTACAG	mCherry-int-a	CACCTTGTAGATGAACTCGCCGT	Verification PCR to confirm integration of mCherry tag at the C-terminus of PHO85, external 5' recombination
Gal7T-s	CTGGTTCCTGCTGGTATTGGCTT	PHO85-3'flank-a1	GAGGATAATGAGAGATGGCAGGCTAAA	Verification PCR to confirm integration of mCherry tag at the C-terminus of PHO85, external 3' recombination
CnPho84_frt	CCTACTCGTTACCGATCAACTG	CnPho84_rrt	AGTCTCGGGAAGAAGCAATG	PHO84 expression
CnPho89_frt	GTGCTCGGTAACAGACTGAC	CnPho89_rrt	ACGCTCGCCAGTTAATCG	PHO89 expression
CnPho840_frt	CCTTCCCGCCGTTATCTAC	CnPho840_rrt	GATACTCGTGTGCCCTACC	PHO840 expression
CnVtc4_frt	GATGCCGTCGGTATGGTTTC	CnVtc4_rrt	TAACAACGCCGCGCAAAG	VTC4 expression

ACT1-RTF	ATGGTATTGCCGACCGTATG	ACT1-RTR	CTCTTCGCGATCCACATCTG	ACT1 expression
HLH3-int-s	TTCAAATCCCGTCCTTCGCCG	HLH3-int-a	CGTTCATAGCGGCAGGAGGCAT	PHO4 expression
APH qRT-s v2	CCTACTTCCCACTCAACCAATCCA	APH qRT-a v2	CCTGCGAAGCCACAAACGAA	APH1 expression
06967qRT L1	TCTCGGTCACTCTGCCTTCT	06967qRT R1	CGCCTTAGCAGGAGCATATC	APH2 expression
02681qRT L1	GACCGATTCTGCTCCTCAAG	02681qRT R1	GGGACGATTCGGGAAAGA	APH3 expression
06115qRT L1	AAAGCAATGCCACGGTAAAC	06115qRT R1	CCCAAGCCTGTAGATTGCAT	APH4 expression
GDE1 s	AAGAAGGTAGGAAGGGAGAGCGG	GDE1 a	ATGAAGATTGACGGGAGACGCC	PHO81 expression
GDE2 s	GGGGTATTGCCAGTGTCATTTCAGA	GDE2 a	TATTCCGTTTCTTCCTTGGCGAG	GDE2 expression
BTA1 s	CCCATTCCAACGCTTTCTACTCTCA	BTA1 a	AGCGACTCATCAGGAAGACCCC	BTA1 expression
PHO80-s	ATAACCTGCGTCAACCTCCCGA	PHO80-a	ATGTCGGTGTCGGCTGGTTCA	PHO80 expression
PHO85-s	CAGCAACAGCAGCAGCAGTATGAGA	PHO85-a	ATCTGTGCGGCTGCTTGAGG	PHO85 expression
PHO81 ots s	ATGTATTATCCTGCTCTGTCGCTCCA	PHO81 5'a	CTCCAGCTCACATCCTCGCAGCAACTGGCTGGAGATAAAGC	PHO81 deletion. Upstream region of PHO81
PHO81 3's	TCCGCCAATCGCCGTATAGAGACATGTAACTGTAATACTAGC	PHO81 ots a	TACGCCAACCTTCCCCTGCTT	PHO81 deletion. Downstream region of PHO81
PHO81 5's	CCCATACTTGCCTTCATACCCTTCAG	PHO81 3'a	GCGATTGATTGATGAGGGATAGGG	Overlap PCR to fuse all 3 fragments for PHO81 deletion
PHO81 ots s	ATGTATTATCCTGCTCTGTCGCTCCA	ActP-a	TGTTGTTACCATCATCCTCTCCTC	Verification PCR to confirm PHO81 deletion, external 5' recombination
Gal7T-s	CTGGTTCCTGCTGGTATTGGCTT	PHO81 ots a	TACGCCAACCTTCCCCTGCTT	Verification PCR to confirm PHO81 deletion, external 3' recombination
PHO81 5's	CCCATACTTGCCTTCATACCCTTCAG	(NEO)PHO81-Rec-5'a	CTCCAGCTCACATCCTCGCAGGGTGCTAATAATAATCTTCC	PHO81 reconstitution. Amplification of PHO81 genomic locus.
PHO81-Rec-5's	ATGTTTGCGATGGGCTGTGTGA	Neo-a	GGAGCCATGAAGATCCTGAGG	Overlap PCR to fuse fragments for reconstitution of PHO81.
SPX-Seq2	CCAATCCACTACTGACCCGAGC	PHO81 3'flank-a	TCTGAACCGGGCGACAATGCT	Verification PCR to confirm ectopic reconstitution of PHO81 gene to *Δpho81:HYGB*
CnPho84_frt	CCTACTCGTTACCGATCAACTG	CnPho84_rrt	AGTCTCGGGAAGAAGCAATG	PHO84 expression
CnPho89_frt	GTGCTCGGTAACAGACTGAC	CnPho89_rrt	ACGCTCGCCAGTTAATCG	PHO89 expression
CnPho840_frt	CCTTCCCGCCGTTATCTAC	CnPho840_rrt	GATACTCGTGTGCCCTACC	PHO840 expression
CnVtc4_frt	GATGCCGTCGGTATGGTTTC	CnVtc4_rrt	TAACAACGCCGCGCAAAG	VTC4 expression
ACT1-RTF	ATGGTATTGCCGACCGTATG	ACT1-RTR	CTCTTCGCGATCCACATCTG	ACT1 expression
HLH3-int-s	TTCAAATCCCGTCCTTCGCCG	HLH3-int-a	CGTTCATAGCGGCAGGAGGCAT	PHO4 expression
APH qRT-s v2	CCTACTTCCCACTCAACCAATCCA	APH qRT-a v2	CCTGCGAAGCCACAAACGAA	APH1 expression
06967qRT L1	TCTCGGTCACTCTGCCTTCT	06967qRT R1	CGCCTTAGCAGGAGCATATC	APH2 expression
02681qRT L1	GACCGATTCTGCTCCTCAAG	02681qRT R1	GGGACGATTCGGGAAAGA	APH3 expression
06115qRT L1	AAAGCAATGCCACGGTAAAC	06115qRT R1	CCCAAGCCTGTAGATTGCAT	APH4 expression
GDE1 s	AAGAAGGTAGGAAGGGAGAGCGG	GDE1 a	ATGAAGATTGACGGGAGACGCC	PHO81 expression
GDE2 s	GGGGTATTGCCAGTGTCATTTCAGA	GDE2 a	TATTCCGTTTCTTCCTTGGCGAG	GDE2 expression
BTA1 s	CCCATTCCAACGCTTTCTACTCTCA	BTA1 a	AGCGACTCATCAGGAAGACCCC	BTA1 expression
PHO80-s	ATAACCTGCGTCAACCTCCCGA	PHO80-a	ATGTCGGTGTCGGCTGGTTCA	PHO80 expression
PHO85-s	CAGCAACAGCAGCAGCAGTATGAGA	PHO85-a	ATCTGTGCGGCTGCTTGAGG	PHO85 expression
GSY2 s	AGCCCGAGAATGAGGACCG	GSY2 a	CAGAAGCAAGAGAGTCCGAGCG	GSY2 expression
GLC3 s	GCTGGAGGACAAATACAAGTGGCT	GLC3 a	GCCAGTTCTTCCGCCCATTC	GLC3 expression
